# Synthesis, Characterization, and *In Vitro* Cytotoxicity of Unsymmetrical Tetradentate Schiff Base Cu(II) and Fe(III) Complexes

**DOI:** 10.1155/2021/6696344

**Published:** 2021-05-03

**Authors:** Quang Trung Nguyen, Phuong Nam Pham Thi, Van Tuyen Nguyen

**Affiliations:** Institute of Chemistry, Vietnam Academy of Science and Technology, 18 Hoang Quoc Viet, Cau Giay, Hanoi, Vietnam

## Abstract

Unsymmetrical tetradentate Schiff base Fe(III) and Cu(II) complexes were prepared by the coordination of some unsymmetrical tetradentate Schiff base ligands with CuCl_2_·2H_2_O or FeCl_3_·6H_2_O. The obtained complexes were characterized by ESI-MS, IR, and UV-Vis. The spectroscopic data with typical signals are in agreement with the suggested molecular formulae of the complexes. Their cyclic voltammetric studies in acetonitrile solutions showed that the Cu(II)/Cu(I) and Fe(III)/Fe(II) reduction processes are at (−)1.882–(−) 1.782 V and at (−) 1.317–(−) 1.164 V, respectively. The *in vitro* cytotoxicity of obtained complexes was screened for KB and Hep-G2 human cancer cell lines. The results showed that almost unsymmetrical tetradentate Schiff base complexes have good cytotoxicity. The synthetic complexes bearing the unsymmetrical tetradentate Schiff base ligands with different substituted groups in the salicyl ring indicate different cytotoxicity. The obtained Fe(III) complexes are more cytotoxic than Cu(II) complexes and relative unsymmetric Schiff base ligands.

## 1. Introduction

Tetradentate salen-type Schiff bases obtained by the condensation of ethylendiamine derivatives with salicylaldehydes are attracted by many researchers because of their synthetic availability and interesting applications including catalytic, biological chemistry [1, 2] and coordination chemistry [3]. Transition metal complexes of tetradentate Schiff base ligands occupy a principal role in coordination chemistry for analysis, catalysis, materials science, and biochemistry [4–7]. Metallosalens are highly versatile coordination compounds with a wide range of bioinorganic and medicinal chemistry such as enzyme mimics, sensing, bioimaging, and medicinal applications [8, 9]. Some metal salen complexes have good DNA binding and RNA cleavage activity [10–12]. They have shown diverse structures and properties generating a variety of stereochemistry and bonding interactions [13, 14]. Besides, the synthesis, characterization, and applications of symmetrical tetradentate Schiff base complexes have been thoroughly studied and reported in the literature [15–17] recently, transition metal complexes with unsymmetrical tetradentate Schiff base ligands were reported for various applications [18–21]. Particularly, some synthetic Cu(II) and Fe(III) complexes with unsymmetrical tetradentate Schiff base ligands show potential DNA binding ability and bioactivity [22–26]. In this study, we continue to describe the synthesis, characterization, and *in vitro* cytotoxicity of the copper(II) and iron(III) complexes with some unsymmetrical tetradentate Schiff bases.

## 2. Materials and Methods

Analytical reagent grade chemicals such as *o*-phenylenediamine (98%) and salicylaldehydes were obtained from Across Organics and used without any purification. All solvents were purified by following the appropriate purification procedures.

Ultra-high performance liquid chromatography combined with hybrid quadrupole time-of-flight tandem mass spectra (UPLC-Q-TOF-MS) of the prepared unsymmetrical tetradentate Schiff base ligands were conducted on an ExionLC AC Series HPLC system coupled with a hybrid quadrupole time-of-flight tandem mass spectrometer (X500R QTOF System) equipped with Turboionspray source. Chromatographic separation was performed on a Kinetex C_18_ column (30 mm × 2.1 mm, 1.7 *µ*m), and the column temperature was maintained at 30°C. The mobile phase consisted of methanol and water containing 0.1% formic acid in gradient mode of 50% methanol for 0–5 min and 100% methanol at 5 min with a flow rate of 0.3 mL·min^−1^. Electrospray ionization mass spectra (m/z) of the synthetic complexes were estimated on Agilent 6310 Ion Trap spectrometer (ESI-MS). Infrared spectra (4000–400 cm^−1^) were recorded on a Perkin Elmer Spectrum Two spectrophotometer using KBr pellet. ^1^H-NMR and ^13^C-NMR spectra were determined in DMSO-d_6_ solution using a Bruker Advance 500 MHz NMR spectrometer with TMS as the internal standard and chemical shifts (*δ*) were recorded in ppm. UV-Visible absorption spectra of the complexes (200–600 nm) were measured in methanol solution (2 × 10^−5^ M) on Perkin Elmer Lambda UV-35 spectrophotometer at room temperature. The effective magnetic measurements of the obtained complexes (*µ*_eff_) were carried out at room temperature using a magnetic susceptibility balance (Mark 1, serial No. 25179) of Sherwood Scientific Ltd.

### 2.1. Synthesis of Unsymmetrical Tetradentate Schiff Base Ligands

Unsymmetrical tetradentate Schiff base ligands were prepared by one-pot method including two-step reactions similarly according to the known procedures [27, 28]. In the first step, monocondensed half-units were prepared by the condensation of *o*-phenylenediamine with 5-*t*-butylsalicylaldehyde or 5-methoxysalicylaldehyde. In the second step, the monocondensed half-unit was mixed with a methanol solution of relative salicylaldehydes. *O*-phenylenediamine (15.5 mmol) dissolved in methylene chloride (25 mL) was added in a 100 ml flask containing 5-*t*-butylsalicylaldehyde or 5-methoxysalicylaldehyde (15.5 mmol) and was stirred for 3 h. After the monocondensed half-units were obtained completely by TLC checking, relative salicylaldehyde (15.5 mmol) in methanol (25 mL) was added and the new mixture was stirred under ultrasound for 1 h more and then the productive precipitates were collected after filtered and washed by cold ethanol. The products were recrystallized from ethyl acetate and dried *in vacuo*.

4-(*tert*-butyl)-2-(*E*)-((2-(*E*)-2-hydroxybenzylidene)amino)phenyl)imino)methyl)phenol (H_2_L1): yellow powder (72%). **Q-TOF-MS** (*m/z*): 373.1909 [M + H]^+^ (Cal.: 373.4675); **IR** (KBr, cm^−1^): 2967 (*v*, C−H), 2748 (*v*, O−H), 1611 (*v*, C=N), 1567 (*v*, C=C), 1484, 1369, 1277 (*v*, C−N), 1186 (*v*, C−O); 823, 758 (*δ*, C-H), 506; ^**1**^**H-NMR** (DMSO-d_6_, 500 MHz, *δ* (ppm), *J* (Hz)): *δ* 12.91 and 12.57 (s, 2H, 2OH), 8.94 and 8.93 (s, 2H, 2HC=N), 7.71–7.65 (m, 2H, 2H-Sal), 7.48–7.45 (m, 3H, 2H-Ph, 1H-Sal), 7.43–7.33 (m, 3H, 2H-Ph, 1H-Sal), 6.99–6.93 (m, 2H, 2H-Sal), 6.90 (d, *J* = 10.0 Hz, 1H, H-Sal), 1.29 (s, 9H, (CH_3_)_3_); ^**13**^**C-NMR** (DMSO-d_6_, 125 MHz, *δ* (ppm)): *δ* 163.97 and 163.83 (2C, 2C=N), 160.38 and 160.31 (2C, 2C-O), 158.03 (1C, C-t-Bu), 142.47 and 142.12 (2C, 2C-Ph), 141.21 (1C, C-Sal), 133.21 (1C, C-Sal), 132.36 (1C, C-Sal), 130.60 (1C, C-Sal), 128.51 (1C, C-Sal), 127.73 and 127.55 (2C, 2C-Ph), 119.55 (1C, C-Sal), 119.41 (1C, C-Sal), 118.95 and 118.82 (2C, 2C-Ph), 116.58 (1C, C-Sal), 116.16 (1C, C-Sal), 33.74 (1C, C(CH_3_)_3_), 30.96 (3C, 3CH_3_); **UV-Vis** (MeOH, 2 × 10^−5^ M, nm, *ε*): 234 (30,000); 275 (24,500); 335 (18,500).

4-(*tert*-butyl)-2-(*E*)-((2-(*E*)-5-fluoro-2-hydroxybenzylidene)amino)phenyl)imino)methyl)phenol (H_2_L2): yellow powder (67%); Q-**TOF-MS** (*m/z*): 391.1799 [M + H]^+^ (Cal. 391.4580); **IR** (KBr, cm^−1^): 2919 (*v*, C−H), 2718 (*v*, O−H), 1615 (*v*, C=N); 1574 (*v*, C=C), 1485, 1356, 1264 (*v*, C−N), 1202 (*v*, C−O), 821, 757 (*δ*, C-H), 509; ^**1**^**H-NMR** (DMSO-d_6_, 500 MHz, *δ* (ppm)): *δ* 12.61 and 12.59 (2H, 2OH), 8.94 and 8.91 (2H, 2HC=N), 7.69 (d, *J* = 2.5, 1H, H-Sal), 7,53 (dd, *J* = 9.0, 3.5, 1H, H-Sal), 7.48–7.39 (m, 5H, 2H-Sal, 3H-Ph), 7.27 (dt, *J* = 9.0, 3.5, 1H, H-Ph), 6.97 (q, *J* = 4.5 Hz, 1H, H-Sal), 6.89 (d, *J* = 8.5, 1H, H-Sal), 1.29 (s, 9H, (CH_3_)_3_)); ^**13**^**C-NMR** (DMSO-d_6_, 125 MHz, *δ* (ppm)): *δ* 163.89 and 162.13 (2C, 2C=N), 158.08 and 156.50 (2C, 2C-O), 155.77 and 155.90 (1C, C-F), 142.39 (1C, C-C((CH_3_)_3_), 142.14 and 141.20 (2C, 2C-Ph), 130.61 (1C, C-Sal), 128.47 (1C, C-Sal), 127.95 and 127.56 (2C, 2C-Ph), 120.33 and 120.14 (2C, 2C-Ph), 119.83 and 119.77 (1C, C-Sal(F)), 119.52 (1C, C-Sal), 118.83 (1C, C-Sal), 118.01 and 117.95 (1C, C-Sal(F)), 116.66 and 116.47 (1C, C-Sal(F)), 116.19 (1C, C-Sal), 33.73 (1C, C-(CH_3_)_3_), 31.11 (3C, 3CH_3_); **UV-Vis** (MeOH, 2 × 10^−5^ M, nm, *ε*): 234 (21,500); 275 (17,500); 340 (13,500).

4-(*tert*-butyl)-2-(*E*)-((2-(*E*)-5-chloro-2-hydroxybenzylidene)amino)phenyl)imino)methyl)phenol (H_2_L3): yellow powder (61%); **Q-TOF-MS** (*m/z*): 429.2555 [M + Na]^+^ (Cal. 429.8941); **IR** (KBr, cm^−1^): 2952 (*v*, C−H), 2697 (br, *v*, O−H), 1614 (*v*, C=N), 1567 (*v*, C=C), 1486, 1354, 1277 (*v*, C−N); 1181 (*v*, C−O), 823, 757 (*δ*, C−H), 506; ^**1**^**H-NMR** (CDCl_3_, 500 MHz, *δ* (ppm)): *δ* 13.09 and 12.74 (2H, 2OH), 8.64 and 8.56 (2H, 2HC=N), 7.43 (dd, *J* = 8.5, 2.5, 1H, H-Sal), 7.36–7.33 (m, 4H, 2H-Ph, 2H-Sal), 7.29 (dd, *J* = 8.5, 2.5, 1H, H-Sal), 7.24–7.22 (m, 2H, 2H-Ph), 7.00 (d, *J* = 7.0, 1H, H-Sal), 6.98 (d, *J* = 7.0, 1H, H-Sal), 1.32 (s, 9H, (CH_3_)_3_); ^**13**^**C-NMR** (CDCl_3_, 125 MHz, *δ* (ppm)): *δ* 164.30 and 162.26 (2C, 2C=N), 159.96 and 159.08 (2C, 2C-O), 142.96 (1C, C-C(CH_3_)_3_), 142.03 and 141.79 (2C, 2C-Ph), 133.09 (1C, C-Sal), 131.19 (1C, C-Sal), 131.09 (1C, 1C-Sal), 128.72 (1C, C-Sal), 128.16 and 127.60 (2C, C-Ph), 123.52 (1C, C-Cl), 119.97 (1C, C-Sal), 119.72 and 119.62 (2C, 2C-Ph), 119.16 (1C, C-Sal), 118.46 (1C, C-Sal), 117.15 (1C, C-Sal), 34.03 (1C, C(CH_3_)_3_), 31.40 (3C, 3CH_3_); **UV-Vis** (MeOH, 2 × 10^−5^ M, nm, *ε*): 235 (28,000); 276 (18,500); 335 (15,000).

4-bromo-2-((*E*)-((2-(((*E*)-5-(*tert*-butyl)-2-hydroxybenzylidene)amino)phenyl)imino)methyl)phenol (H_2_L4): yellow powder (56%); **Q-TOF-MS** (*m/z*): 451,1032 [M + H]^+^ (Cal. 452.3636); **IR** (KBr, cm^−1^): 2953 (*v*, C−H), 2678 (br, *v*, O−H), 1612 (*v*, C=N), 1561 (*v*, C=C), 1474, 1354, 1276 (*v*, C−N), 1181 (*v*, C−O), 821, 756 (*δ*, C−H), 507; ^**1**^**H-NMR** (DMSO-*d*_*6*_, 500 MHz, *δ* (ppm)): *δ* 13.03 and 12.51 (2H, 2OH), 8.94 and 8.91 (2H, 2HC=N), 7.89 (d, *J* = 2.0, 1H, H-Sal), 7.71 (d, *J* = 2.5, 1H, H-Sal), 7.54 (dd, *J* = 9.0, 2.5, 1H, H-Sal), 7.47–7.40 (m, 5H, 2H-Ph, 3H-Sal), 6.93 (d, *J* = 8.5, 1H, H-Ph), 6.88 (d, *J* = 8.5, 1H, H-Ph), 1.29 (s, 9H, (CH_3_)_3_); ^**13**^**C-NMR** (DMSO-*d*_*6*_, 125 MHz, *δ* (ppm)): *δ* 163.88 and 162.14 (2C, 2C=N)), 159.49 and 158.01 (2C, 2C-O), 142.53 (1C, C-C((CH_3_)_3_), 141.80 and 141.24 (2C, 2C-Ph), 135.53 (1C, C-Sal), 133.78 (1C, C-Sal), 130.65 (1C, C-Sal), 128.39 and 128.06 (2C, 2C-Ph), 127.54 (1C, C-Sal), 121.32 (1C, C-Sal), 119.60 and 119.54 (2C, 2C-Ph), 119.06 (1C, C-Sal), 118.87 (1C, C-Sal), 116.18 (1C, C-Sal), 109.72 (1C, C-Br), 33.74 (1C, C(CH_3_)_3_); 31.11 (3C, 3CH_3_); **UV-Vis** (MeOH, 2 × 10^−5^ M, nm, *ε*): 235 (26,000); 275 (17,500); 337 (14,000).

2-((*E*)-((2-(((*E*)-2-hydroxy-3-methoxybenzylidene)amino)phenyl)imino)methyl)-4-methoxyphenol (H_2_L5): orange powder (53%); **Q-TOF-MS** (*m/z*): 377,1482 [M + H]^+^ (Cal. 377.4132); **IR** (KBr, cm^−1^): 2936 (*v*, C−H), 2737 (br, *v*, O−H), 1611 (*v*, C=N), 1578 (*v*, C=C), 1464, 1364, 1271 (*v*, C−N), 1212 (*v*, C−O), 1041, 818, 740 (*δ*, C−H), 481; ^**1**^**H-NMR** (DMSO-*d*_*6*_, 500 MHz, *δ* (ppm)): *δ* 13.30 and 12.00 (2H, 2OH), 8.93 and 8.90 (2H, 2HC=N), 7.49 (m, 1H, H-Ph), 7.43–7.38 (m, 3H, 3H-Ph), 7.34 (d, *J* = 3.5, 1H, H-Sal), 7.23 (dd, *J* = 8.0, 1.0, 1H, H-Sal), 7.10 (dd, *J* = 8.0, 1.5, 1H, H-Sal), 7.03 (dd, *J* = 9.0, 3.0, 1H, H-Sal), 6.90 (d, *J* = 9.5, 1H, 1H-Sal), 6.89 (t, *J* = 7.5, 1H, H-Sal), 3.80 (s, 3H, OCH_3_), 3.76 (s, 3H, OCH_3_); ^**13**^**C-NMR** (DMSO-*d*_*6*_, 125 MHz, *δ* (ppm): *δ* 163.65 and 162.62 (2C, 2C=N), 154.21 (1C, C−OH); 151.92 (1C, C−OH), 151.20 (1C, C−OMe), 148.00 (1C, C−OMe), 142.66 and 141.68 (2C, 2C-Ph), 127.84 and 127.63 (2C, 2C-Ph), 123.76 (1C, C-Sal), 120.82 (1C, C-Sal), 119,69 (1C, C-Sal), 119.47 (2C, C-Sal), 119.25 (1C, C-Sal), 118.33 and 117,54 (2C, 2C-Ph), 115.45 (1C, C-Sal), 114.38 (1C, C-Sal), 55.71 (1C, O-CH_3_), 55.54 (1C, O-CH_3_); **UV-Vis** (MeOH, 2 × 10^−5^ M, nm, *ε*): 232 (27,500); 279 (20,500); 340 (14,500).

### 2.2. Preparation of Unsymmetrical Schiff Base Complexes

Unsymmetrical Schiff base complexes were prepared from the coordination between the obtained unsymmetrical Schiff base ligands and CuCl_2_·2H_2_O or FeCl_3_·6H_2_O in a molecular ratio 1 : 1. 1.0 mmol CuCl_2_·2H_2_O or FeCl_3_·6H_2_O dissolved in ethanol was added to an ethanol solution of 1.0 mmol ligand. The reaction mixtures were refluxed at the presence of 1.0 mmol Na_2_CO_3_ for 3 hrs; then, the reaction mixtures were cooled to room temperature. The productive precipitates were collected after filtered and washed by cold ethanol and then dried in vacuo.

[Cu(II)L1]: dark brown powder, 91%; **ESI-MS** (*m/z*): 433.9 [M + H]^+^ (Cal. 434.9); **IR** (KBr, cm^−1^): 2954 (*v*, C−H), 1607 (*v*, C=N), 1521, 1458 (*v*, C=C), 1379, 1257 (*v*, C−N), 1182 (*v*, C−O), 1148, 832, 745 (*δ*, C−H), 530 (Cu−O), 430 (Cu−N); **UV-Vis** (MeOH, 2 × 10^−5^ M, nm, *ε*): 249 (22,500), 310 (15,500), 343 (11,500), 424 (10,000); *µ*_eff_ = 1.81 BM.

[Cu(II)L2]: pale yellow solid, 92%; **ESI-MS** (*m/z*): 451.9 [M + H]^+^ (Cal. 452.9); **IR** (KBr, cm^−1^): 2955 (*v*, C−H), 1619 (*v*, C=N), 1525, 1462 (*v*, C=C), 1382, 1258 (*v*, C−N), 1179 (*v*, C−O), 1149, 829, 743 (*δ*, C−H), 531 (Cu−O), 430 (Cu−N); **UV-Vis** (MeOH, 2 × 10^−5^ M, nm, *ε*): 249 (25,000), 300 (18,000), 343 (13,500), 425 (10,500); *µ*_eff_ = 1.97 BM.

[Cu(II)L3]: pale yellow powder, 92%; **ESI-MS** (*m/z*): 467.9 [M + H]^+^ (Cal. 469.4); **IR** (KBr, cm^−1^): 2957 (*v*, C−H), 1616 (*v*, C=N), 1524, 1456 (*v*, C=C), 1379, 1259 (*v*, C−N), 1179 (*v*, C−O), 1109, 824, 746 (*δ*, C−H), 538 (Cu−O), 433 (Cu−N); **UV-Vis** (MeOH, 2 × 10^−5^ M, nm, *ε*): 250 (34,000), 301 (20,500), 342 (15,000), 425 (13,500); *µ*_eff_ = 1.96 BM.

[Cu(II)L4]: brown powder, 91%; **ESI-MS** (*m/z*): 513.9 [M + H]^+^ (Cal. 513.9); **IR** (KBr, cm^−1^): 2962 (*v*, C−H), 1617 (*v*, C=N), 1518, 1461 (*v*, C=C), 1384, 1257 (*v*, C−N), 1172 (*v*, C−O), 811, 755 (*δ*, C−H); 522 (Cu−O), 427 (Cu−N); **UV-Vis** (MeOH, 2 × 10^−5^ M, nm, *ε*): 250 (28,500), 301 (17,500), 342 (13,000), 426 (11,500); *µ*_eff_ = 2.00 BM.

[Cu(II)L5]: red brown powder, 90%; **ESI-MS** (*m/z*): 437.9 [M + H]^+^ (Cal. 437.9); **IR** (KBr, cm^−1^) 2928 (*v*, C−H), 1604 (*v*, C=N), 1531, 1473 (*v*, C=C), 1369, 1222 (*v*, C−N), 1194 (*v*, C−O), 1160, 818, 742 (*δ*, C−H), 528 Cu−O), 411 (Cu−N); **UV-Vis** (MeOH, 2 × 10^−5^ M, nm, *ε*): 245 (30,500), 318 (16,500), 356 (12,500), 440 (7,500); *µ*_eff_ = 1.94 BM.

[Fe(III)L1Cl]: dark brown powder (89%); **ESI-MS** (*m/z*): 425.9 [M–Cl]^−^ (Cal. 426.2); **IR** (KBr, cm^−1^): 2951 (*v*, C-H), 1602 (*v*, C=N), 1528 (*v*, C=C), 1461, 1375, 1258 (*v*, C‒N), 1190 (*v*, C‒O), 1149, 813, 747 (*δ*, C‒H); 613, 536 (Fe‒O); 475 (Fe‒N); UV-Vis (MeOH, 2 × 10^−5^ M, nm, *ε*): 246 (28,000), 297 (34,500), 372 (16,500), 424 (10,500); *µ*_eff_ = 5.98 BM.

[Fe(III)L2Cl]: red brown powder (93%); **ESI-MS** (*m/z*): 443.9 [M–Cl]^−^ (Cal. 444.2); **IR** (KBr, cm^−1^): 2951 (*v*, C-H), 1611 (*v*, C=N), 1530 (*v*, C=C), 1464, 1376, 1254 (*v*, C‒N), 1179 (*v*, C‒O), 1143, 828, 758 (*δ*, C‒H); 532 (Fe‒O); 482 (Fe‒N); **UV-Vis** (MeOH, 2 × 10^−5^ M, nm, *ε*): 246 (26,000), 297 (32,000), 375 (14,500), 427 (10,000); *µ*_eff_ = 5.96 BM.

[Fe(III)L3Cl]: brown solid powder (92%); **ESI-MS** (*m/z*): 459.9 [M–Cl]^−^ (Cal. 460.7); **IR** (KBr, cm^−1^): 2964 (*v*, C−H), 1610 (*v*, C=N), 1527 (*v*, C=C), 1453, 1378, 1260 (*v*, C−N), 1181 (*v*, C‒O), 826, 760 (*δ*, C‒H), 669, 535 (Fe‒O), 479 (Fe‒N); **UV-Vis** (MeOH, 2 × 10^−5^ M, nm, *ε*): 247 (31,500), 297 (35,500), 375 (15,500), 424 (11,500); *µ*_eff_ = 5.99 BM.

[Fe(III)L4Cl]: brown solid powder (90%); **ESI-MS** (*m/z*): 503.9 [M–Cl]^−^ (Cal. 505.1); **IR** (KBr, cm^−1^): 2961 (*v*, C−H), 1604 (*v*, C=N), 1523 (*v*, C=C), 1449, 1376, 1258 (*v*, C−N), 1183 (*v*, C‒O), 824, 759 (*δ*, C‒H), 650, 534 (Fe‒O), 476 (Fe‒N); **UV-Vis** (MeOH, 2 × 10^−5^ M, nm, *ε*): 243 (35,000), 304 (29,500), 382 (15,500), 426 (9,500); *µ*_eff_ = 5.90 BM.

[Fe(III)L5Cl]: dark brown solid powder (85%); **ESI-MS** (*m/z*): 429.9 [M–Cl]^−^ (Cal. 430.2); **IR** (KBr, cm^−1^): 2925 (*v*, C−H), 1599 (*v*, C=N), 1533 (*v*, C=C), 1461, 1378, 1251 (*v*, C−N), 1185 (*v*, C‒O), 820, 734 (*δ*, C‒H), 577, 535 (Fe‒O), 415 (Fe‒N); **UV-Vis** (MeOH, 2 × 10^−5^ M, nm, *ε*): 242 (30,000), 305 (34,500), 390 (13,500), 452 (8,000); *µ*_eff_ = 5.94 BM.

### 2.3. Electrochemical Studies

The electrochemical studies of all complexes were performed using Zahner IM6 instrument. The cyclic voltammograms of Cu(II) complexes and Fe(III) complexes were recorded using 1.0 × 10^−3^ M concentration in acetonitrile solution and 0.1 M LiClO_4_ as supporting electrolyte. The working electrode was platinum wire which was polished, washed, and dried. The reference electrode was Ag/AgCl/KCl and platinum wire was the counter electrode. All experiments were performed in standard electrochemical cells at room temperature at a scan rate of 100 mV·s^−1^ with the potential window −3 V to +3 V *vs* Ag/AgCl/KCl reference electrode.

### 2.4. *In Vitro* Cytotoxicity

MTT (3-(4,5-dimethylthiazol-2-yl)-2,5-diphenyltetrazolium) method was used to estimate *in vitro* cytotoxicity of obtained ligands and synthetic complexes. Human cancer cells KB and Hep-G2 were cultured in DMEM with 10% fetal bovine serum, 100 *µ*g/mL streptomycin, 100 units/mL penicillin, and 2 mmol/L L-glutamine at 37°C in a humidified atmosphere with 5% CO_2_ and 95% air. Cancer cells were cultivated in 96-well plates for 24 hrs followed by treating with different concentrations of complexes in DMSO and incubated continuously for 48 hrs more. Then, testing cells were exposed to 10 *µ*L of freshly prepared MTT (5 mg/ml) solution and incubated for 4 h at 37°C in an atmosphere of 5% CO_2_. The formazan crystals obtained during MTT incubation were dissolved in 100 *µ*L of DMSO. The absorbance was recorded at 540 nm on Genios TECAN spectrophotometer. The experiments were carried out in triplicate for every concentration of the complexes. The percent viable cells were plotted as a function of concentration to determine the IC_50_ values presented in Table 1.

## 3. Results and Discussion

### 3.1. Synthesis and Characterization

**Scheme 1:**
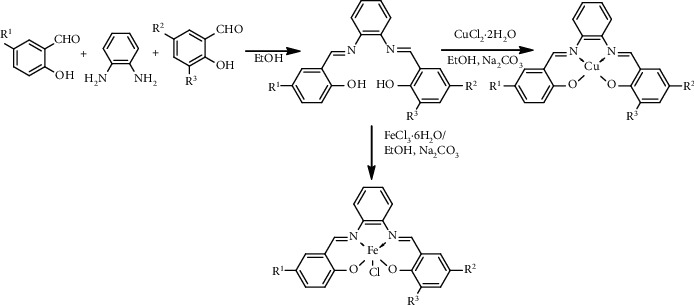
Synthesis of unsymmetrical tetradentate Schiff base ligands and their Cu(II) and Fe(III) complexes.

The unsymmetrical tetradentate Schiff base ligands (H_2_L1–H_2_L5) (Table 2) were synthesized following a one-pot procedure in moderate yields (53–72%) (Scheme 1) and high purity (>98.5%) performed by UPLC (supplementary data). The obtained ligands are soluble in organic solvents such as ethanol, ethyl acetate, and dichloromethane. These compounds were characterized by Q-TOF-MS, IR, ^1^H-NMR, ^13^C-NMR, and UV-Vis spectroscopies. Cu(II) and Fe(III) complexes were prepared following the coordination of CuCl_2_·2H_2_O or FeCl_3_·6H_2_O with each obtained ligand in good yields (85–93%) in ethanol (Scheme 1). The synthetic unsymmetrical tetradentate Schiff base complexes are soluble in DMSO, acetonitrile, methanol, and dichloromethane. These complexes were also characterized by ESI-MS, IR, and UV-Vis spectroscopies.

In the high resolution mass spectra, Q-TOF-MS, the pseudo-molecular ion signals of the obtained unsymmetrical ligands are observed as [M + H]^+^ or [M + Na]^+^ which clearly indicate molecular masses suitable for the suggested formulae. In ESI-MS spectra of synthetic complexes, pseudo-molecular ion peaks are observed as [M + H]^+^ for Cu(II) complexes and [M–Cl]^−^ for Fe(III) complexes. They are quite good in agreement with the suggested formulae (Table 2).

^1^H-NMR spectra of synthetic unsymmetrical tetradentate ligands have typical signals at 13.30–12.00 ppm for two different OH groups and 8.94–8.56 ppm for couples of different HC=N groups which showed obvious evidence for unsymmetrical property of obtained ligands. There are typical signals as single signals at 1.32–1.29 ppm for 9 protons of C(CH_3_)_3_ groups for H_2_L1–H_2_L4 and single signals at 3.80 and 3.76 ppm for 2 different methoxy groups of H_2_L5. In ^13^C-NMR of these tetradentate Schiff base ligands, there are also typical signals at 164.30–162.13 ppm for two different C=N groups, at 160.38–151.92 ppm for two different C−O groups. They are obvious evidences for the unsymmetrical property of synthetic tetradentate ligands. They are characteristic signals at 34.03–33.73 ppm and 31.40–30.96 ppm for carbon signals of *t*-Bu groups of H_2_L1–H_2_L4, at 55.71 and 55.54 ppm, for 2 different methoxy groups of H_2_L5.

In IR spectra, there are typical signals for the formation of ligands at 1615–1611 cm^−1^ of the stretching vibrations (*v*) of C=N bondings. The typical signals at 2748–2678 cm^−1^ belong to *v*_(O−H),_ at 1277–1264 cm^−1^ of *v*_(C−N)_ and at 1212–1181 cm^−1^ of *v*_(C−O)_. IR spectra of obtained Cu(II) complexes possess characteristic signals at 1619–1604 cm^−1^ of *v*_(C=N)_, at 1259–1222 cm^−1^ of *v*_(C−N)_, and at 1179–1148 cm^−1^ of *v*_(C−O)_. There are new signals at 538–522 cm^−1^ and 504–411 cm^−1^ for the stretching vibrations of Cu−N and Cu−O coordination bondings, respectively. In IR spectra of synthetic Fe(III) complexes, there are typical signals at 1611–1599 cm^−1^ of *v*_(C=N)_, at 1260–1251 cm^−1^ of *v*_(C−N)_, and 1185–1143 cm^−1^ of *v*_(C−O)_. New signals are found at 536–532 cm^−1^ and 482–415 cm^−1^ for new bonding vibrations of Fe−N and Fe−O, respectively. The disappearance of O−H signals and the new formation of M−N and M−O are the obvious evidence of the coordination of center metals with obtained ligands through the nitrogen atoms of azomethine groups and oxygen atoms of the phenolic groups (Table 3).

### 3.2. Electronic Spectra and Magnetic Moments

**Figure 1:**
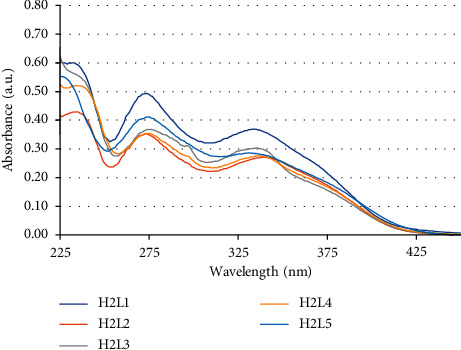
UV-Vis absorption spectra of unsymmetrical tetradentate Schiff base ligands.

On the UV-Vis spectra of the obtained ligands there were three main absorption bands with maximum absorption wavelengths (*λ*_abs_) at about 235 nm (42,553 cm^−1^) assigned to the *π* ⟶ *π*^*∗*^ electronic transitions of the aromatic rings, at about 275 nm (36,364 cm^−1^) and 335 nm (29,851 cm^−1^) attributed to *n* ⟶ *π*^*∗*^ electronic transitions associated with the transfer of lone pair situated at N and O of C=N and C−O groups, respectively [25]. There is a little difference between UV-Vis spectra of these unsymmetrical tetradentate ligands H_2_L1–H_2_L5 (Figure 1). Upon complexation, *n* ⟶ *π*^*∗*^ transition of ligand shifts to a longer wavelength; this indicates the coordination of ligand to metal [26].

**Figure 2:**
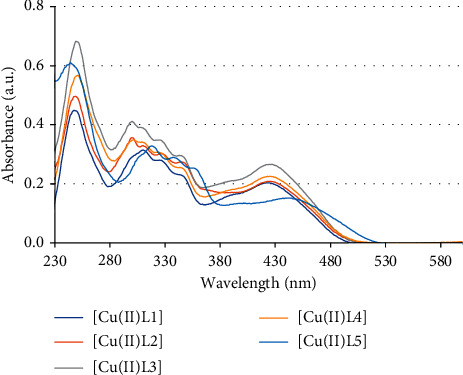
UV-Vis absorption spectra of unsymmetrical Schiff base Cu(II) complexes.

In UV-Vis spectra of unsymmetrical tetradentate Schiff base Cu(II) complexes, besides the main absorption bands of interligand charge transfer transitions with *λ*_abs_ at about 245–250 nm and 300–360 nm (*n* ⟶ *π*^*∗*^), a new broad low-energy absorption band with *λ*_abs_ is observed at 380–500 nm which can be assigned to ligand-to-metal charge transfer (LMCT) and metal-to-ligand (MLCT) transitions [29, 30]. The d-d bands were not observed due to the low concentration (2 × 10^−5^ M) of the solutions. These bands should be low intensity in the region of 550–650 nm. UV-Vis absorption bands of [Cu(II)L1] − [Cu(II)L4] complexes are similar except UV-Vis absorption bands of [Cu(II)L5] complex which are shifted to a longer wavelength region due to the electron-donating property of the substituted methoxy groups (Figure 2).

Magnetic measurements and electronic spectra were conducted in order to obtain information about geometry of the complexes. Copper(II) complexes, in the present study, show *µ*_eff_ values 1.81–2.00 BM which were consistent with presence of one unpaired electron. This behavior suggests square-planar geometry for the copper(II) complexes [31, 32].

**Figure 3:**
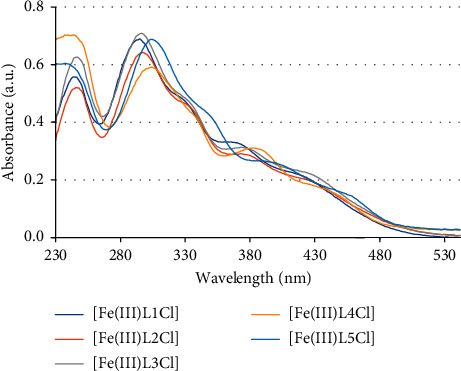
UV-Vis absorption spectra of unsymmetrical tetradentate Schiff base Fe(III) complexes.

In UV-Vis spectra of unsymmetrical tetradentate Schiff base Fe(III) complexes, besides the main absorption bands with wavelength maximum at 242–247 nm, 297–305 nm and a shoulder at 372–390 nm which may be assigned to interligand charge transfer transitions (*n* ⟶ *π*^*∗*^), there is a new broad low-energy absorption band with *λ*_abs_ at 375–485 nm which can belong to LMCT and MLCT transitions. The d-d bands were also not observed due to the low concentration (2 × 10^−5^ M) of the solutions. While UV-Vis absorption bands of [Fe(III)L1Cl] - [Fe(III)L4Cl] complexes are similar, UV-Vis absorption bands of [Fe(III)L5Cl] complex are also moved to a longer wavelength region when it contains the electron-donating methoxy groups reasonably (Figure 3).

Fe(III) complexes exhibit magnetic moments of 5.90–5.99 BM due to the presence of five unpaired electrons, which indicate an octahedral geometry around Fe(III) ions [21, 25].

### 3.3. Electrochemical Studies

**Figure 4:**
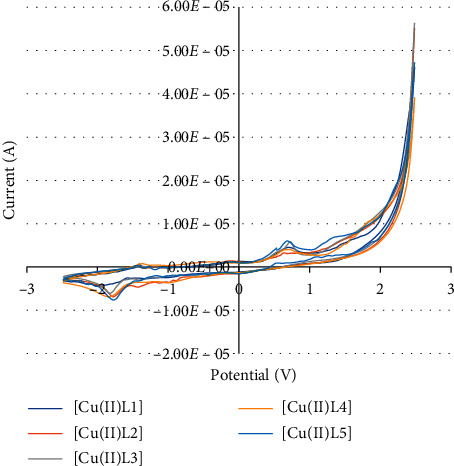
Cyclic voltammograms of unsymmetrical tetradentate Schiff base Cu(II) complexes.

The electrochemical behaviors of the synthetic unsymmetrical tetradentate Schiff base Cu(II) and Fe(III) complexes were investigated using cyclic voltammetry (CV). Cyclic voltammograms were recorded using a Zahner-elektrik IM6 instrument with a standard three-electrode setup, a platinum working electrode, a platinum wire as the counter electrode, and Ag/AgCl/KCl as the reference electrode, at room temperature with voltage scan rate = 100 mV·s^−1^. The concentration of complexes in acetonitrile was 1.0 × 10^−3^ M and 0.1 M LiClO_4_ was used as supporting electrolyte. The cyclic voltammetric profile of synthetic Cu(II) complexes is given in Figure 4. Interestingly, the CVs of synthetic Cu(II) complexes show cathodic peaks at (−)1.882–(−) 1.782 V for the reduction of Cu(II) ⟶ Cu(I). A similar type of cathodic response was found in reported Cu(II) complexes [33]. Some slight differences in the reduction potentials of these Cu(II) complexes should be attributed to the effects of the electron-donating methoxy and electron-withdrawing halogen substituted groups (Table 4).

**Figure 5:**
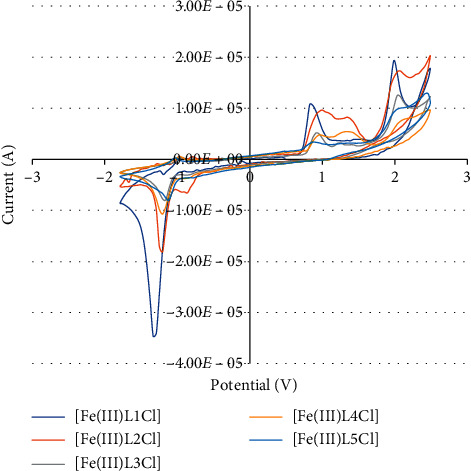
Cyclic voltammograms of unsymmetrical tetradentate Schiff base Fe(III) complexes.

Similarly, the cyclic voltammograms of synthetic Fe(III) complexes are given in Figure 5. Synthetic Fe(III) complexes possess well-defined cathodic peaks at (−) 1.317–(−) 1.164 V for the reduction of Fe(III) ⟶ Fe(II) probably. A similar type of cathodic signals was observed in the reported Fe(III) complexes [34]. The reduction progresses of these Fe(III) complexes are seemingly taken easier than the ones of the Cu(II) complexes. Some difference in the reduction potentials of the Fe(III) complexes must be expected from the electronic effects of the electron-donating and electron-withdrawing substituted groups (Table 4).

### 3.4. *In Vitro* Cytotoxicity Assay

The cytotoxicity of relative unsymmetric ligands, obtained Cu(II) complexes, and Fe(III) complexes against KB and Hep-G2 human cancer cells was determined by MTT-dye reduction method as the standard bioassay using ellipticine as the standard compound for comparison purposes. The bioassay results are presented in Table 1.

The results showed that the synthetic Cu(II) complexes have good cytotoxicity for KB and Hep-G2 (IC_50_ < 100 *µ*M) except [Cu(II)L5] and better than the relative ligands. The synthetic complexes with different substituted groups possess different anticancer activity. The cytotoxic activity order of Cu(II) complexes follows as [Cu(II)L1] > [Cu(II)L2] > [Cu(II)L3] > [Cu(II)L4] > [Cu(II)L5]. The obtained Fe(III) complexes have very excellent cytotoxicity for KB and Hep-G2 (IC_50_ < 20 *µ*M), much better than Cu(II) complexes because Fe(III) complexes with the electrochemical reduction potentials of (−) 1.317–(−) 1.164 V can carry out the redox reaction easier than Cu(II) complexes with the electrochemical reduction potentials of (−)1.882–(−) 1.782 V probably. Fe(III) complexes exhibit an octahedral geometry around central metal ions while copper(II) complexes behave in a square-planar geometry. The cytotoxic activity order of Fe(III) complexes is [Fe(III)L1Cl] > [Fe(III)L5Cl] > [Fe(III)L2Cl]∼[Fe(III)L3Cl] > [Fe(III)L4Cl]. The substituted groups in salicyl rings have some effects to the complexes including their electrochemical properties and their bulk. [Fe(III)L1Cl] without substituted group in the second salicyl ring has the best cytotoxic activity for KB and Hep-G2 with IC_50_ = 0.68 and 0.83 *µ*M, respectively, even better than the standard compound, ellipticine, with IC_50_ = 1.14 and 2.11 *µ*M for KB and Hep-G2, respectively.

## 4. Conclusions

Series of Cu(II) and Fe(III) complexes with unsymmetrical tetradentate Schiff base ligands were synthesized in good yields and characterized by ESI-MS, IR, UV-Vis, and CV spectroscopies. The characteristic spectra of ligands have changed when the coordination of the ligands to metals was carried out. The electron-donating and electron-withdrawing substituted groups of ligands have some effects on their spectral properties. The strong UV-Vis absorption bands for MLCT of the Cu(II) complexes were observed at 422–440 nm, while the weak UV-Vis absorption bands for MLCT of the Fe(III) complexes were observed at 425–450 nm. The obtained copper(II) complexes with *µ*_eff_ values 1.81–2.00 BM show square-planar geometry for the copper(II) complexes. The Fe(III) complexes with magnetic moments of 5.90–5.99 BM indicate an octahedral geometry around Fe(III) ions. Interestingly, the CVs of synthetic Cu(II) complexes show cathodic peaks at (−)1.882–(−) 1.782 V for Cu(II) ⟶ Cu(I) reduction when the obtained Fe(III) complexes possess well-defined cathodic peaks at (−) 1.317–(−) 1.164 V for Fe(III) ⟶ Fe(II) reduction. The cytotoxicity *in vitro* for human cancer cells KB and Hep-G2 was estimated for synthetic Cu(II) complexes, Fe(III) complexes, and the relative ligands. The results showed that the synthetic Cu(II) complexes have good cytotoxicity for KB and Hep-G2 (IC_50_ < 100 *µ*M) except [Cu(II)L5]. The obtained Fe(III) complexes have excellent cytotoxicity for KB and Hep-G2 (IC_50_ < 20 *µ*M), much better than Cu(II) complexes and the relative ligands. Among them, [Fe(III)L1Cl] showed the best cytotoxic activity for KB and Hep-G2 with IC_50_ = 0.68 and 0.83 *µ*M, respectively, better than the standard compound, ellipticine with IC_50_ = 1.14 and 2.11 *µ*M for KB and Hep-G2, respectively.

## Figures and Tables

**Table 1:** *In vitro* cytotoxicity of the unsymmetrical tetradentate Schiff base Cu(II) complexes and Fe(III) complexes.

Compound	IC_50_ (*µ*M)	
	KB	Hep-G2
H_2_L1	>100	>100
[Cu(II)L1]	14.71 ± 0.11	21.04 ± 1.08
[Fe(III)L1Cl]	0.68 ± 0.05	0.83 ± 0.05
H_2_L2	>100	>100
[Cu(II)L2]	46.46 ± 2.42	18.05 ± 0.09
[Fe(III)L2Cl]	3.25 ± 0.16	7.05 ± 0.25
H_2_L3	80.49 ± 0.76	38.32 ± 1.28
[Cu(II)L3]	70.60 ± 3.29	35.99 ± 0.17
[Fe(III)L3Cl]	1.84 ± 0.10	6.07 ± 0.22
H_2_L4	>100	51.08 ± 2.42
[Cu(II)L4]	85.96 ± 4.57	50.52 ± 0.26
[Fe(III)L4Cl]	2.76 ± 0.17	19.78 ± 1.07
H_2_L5	>100	>100
[Cu(II)L5]	96.71 ± 5.11	>100
[Fe(III)L5Cl]	1.95 ± 0.13	2.38 ± 0.17
**Ellipticine**	1.14 ± 0.06	2.11 ± 0.12

**Table 2:** Unsymmetrical tetradentate Schiff base ligands and their Cu(II) and Fe(III) complexes.

R^1^	R^2^	R^3^	Ligand	Cu(II) complex	Fe(III) complex
t-Bu	H	H	H_2_L1	[Cu(II)L1]	[Fe(III)L1Cl]
t-Bu	F	H	H_2_L2	[Cu(II)L2]	[Fe(III)L2Cl]
t-Bu	Cl	H	H_2_L3	[Cu(II)L3]	[Fe(III)L3Cl]
t-Bu	Br	H	H_2_L4	[Cu(II)L4]	[Fe(III)L4Cl]
OCH_3_	H	OCH_3_	H_2_L5	[Cu(II)L5]	[Fe(III)L5Cl]

**Table 3:** Selected typical signals of IR (cm^−1^) spectra of the obtained ligands and synthetic complexes.

Compound	*v*_(O−H)_	*v*_(C=N)_	*v*_(C−N)_	*v*_(C−O)_	*δ*_(O−H)_	*v*_(M−N)_	*v*_(M−O)_
H_2_L1	2748	1611	1277	1187	758	—	—
[Cu(II)L1]	—	1607	1257	1148	745	530	503
[Fe(III)L1Cl]	—	1602	1258	1149	747	536	475
H_2_L2	2718	1615	1264	1202	757	—	—
[Cu(II)L2]	—	1619	1258	1149	743	531	504
[Fe(III)L2Cl]	—	1611	1254	1143	742	532	482
H_2_L3	2697	1614	1277	1181	757	—	—
[Cu(II)L3]	—	1616	1259	1179	746	538	504
[Fe(III)L3Cl]	—	1610	1260	1145	760	535	479
H_2_L4	2678	1612	1276	1181	756	—	—
[Cu(II)L4]	—	1617	1257	1172	755	522	504
[Fe(III)L4Cl]	—	1604	1258	1145	759	534	476
H_2_L5	2737	1611	1271	1212	740	—	—
[Cu(II)L5]	—	1604	1222	1160	742	528	411
[Fe(III)L5Cl]	—	1599	1251	1185	734	535	415

**Table 4:** Reduction potentials of synthetic Cu(II) and Fe(III) complexes.

Complex	*E*pc (V)
[Cu(II)L1]	−1.882
[Cu(II)L2]	−1.782
[Cu(II)L3]	−1.817
[Cu(II)L4]	−1.832
[Cu(II)L5]	−1.782
[Fe(III)L1Cl]	−1.317
[Fe(III)L2Cl]	−1.183
[Fe(III)L3Cl]	−1.195
[Fe(III)L4Cl]	−1.203
[Fe(III)L5Cl]	−1.164

## Data Availability

The data used to support the findings of this study are included within the article and the supplementary information file.

## Supplementary Materials

Supplementary Materials This section contains the spectral data of synthetic unsymmetrical tetradentate Schiff base ligands, Cu(II) and Fe(III) complexes. The spectral data of synthetic unsymmetrical tetradentate Schiff base ligands consist of UPLC, Q-TOF-MS, IR, UV-Vis, ^1^H-NMR, and ^13^C-NMR spectra. The spectral data of the obtained Cu(II) and Fe(III) complexes include ESI-MS, IR, UV-Vis, and CV spectra.
