# Supplementary material for: Synthesis, Characterization, and In Vitro Cytotoxicity of Unsymmetrical Tetradentate Schiff Base Cu(II) and Fe(III) Complexes
Source: Bioinorg Chem Appl. 2021 May 3;2021:6696344. doi: 10.1155/2021/6696344 (PMC8118743; doi:10.1155/2021/6696344)
Supplement: Supplementary Materials — This section contains the spectral data of synthetic unsymmetrical tetradentate Schiff base ligands, Cu(II) and Fe(III) complexes. The spectral data of synthetic unsymmetrical tetradentate Schiff base ligands consist of UPLC, Q-TOF-MS, IR, UV-Vis, 1H-NMR, and 13C-NMR spectra. The spectral data of the obtained Cu(II) and Fe(III) complexes include ESI-MS, IR, UV-Vis, and CV spectra. [file 6696344.f1.docx]

**Supplementary information**

# **Synthesis, Characterization and *In Vitro* Cytotoxicity of Unsymmetrical Tetradentate Schiff base Cu(II) and Fe(III) Complexes**

Quang Trung Nguyen*, Phuong Nam Pham Thi and Van Tuyen Nguyen

Institute of Chemistry, Vietnam Academy of Science and Technology

18 Hoang Quoc Viet, Cau Giay, Hanoi City, Vietnam.

*Corresponding author e-mail address: [trungquang_cnhh@yahoo.com](mailto:trungquang_cnhh@yahoo.com)

**Table of Contents**

1. **The spectroscopies of unsymmetrical tetradentate Schiff base ligands…….…..2**

I.1. The spectra of ligand H_2_L1………………...........................................................2

I.2. The spectra of ligand H_2_L2………………...........................................................4

I.3. The spectra of ligand H_2_L3………………...........................................................7

I.4. The spectra of ligand H_2_L4………………...........................................................9

I.5. The spectra of ligand H_2_L5………………..........................................................12

1. **The spectroscopies of Cu(II) complexes……………………………………….....14**

II.1. The spectra of complex [Cu(II)(L1)]………………..........................................14

II.2. The spectra of complex [Cu(II)(L2)]………………..........................................16

II.3. The spectra of complex [Cu(II)(L3)]..................................................................17

II.4. The spectra of complex [Cu(II)(L4)]…………………......................................19

II.5. The spectra of complex [Cu(II)(L5)]..................................................................20

1. **The spectroscopies of Fe(III) complexes……………..…………………………...22**

III.1. The spectra of complex [Fe(III)(L1)Cl]………………....................................22

III.2. The spectra of complex [Fe(III)(L2)Cl]............................................................23

III.3. The spectra of complex [Fe(III)(L3)Cl]............................................................25

III.4. The spectra of complex [Fe(III)(L4)Cl]………………....................................26

III.5.The spectra of complex [Fe(III)(L5)Cl].............................................................28

1. **The UV-Vis spectra of synthetic compounds…………………………………….29**
2. **The spectroscopies of unsymmetrical tetradentate Schiff base ligands**

I.1. The spectra of ligand H_2_L1


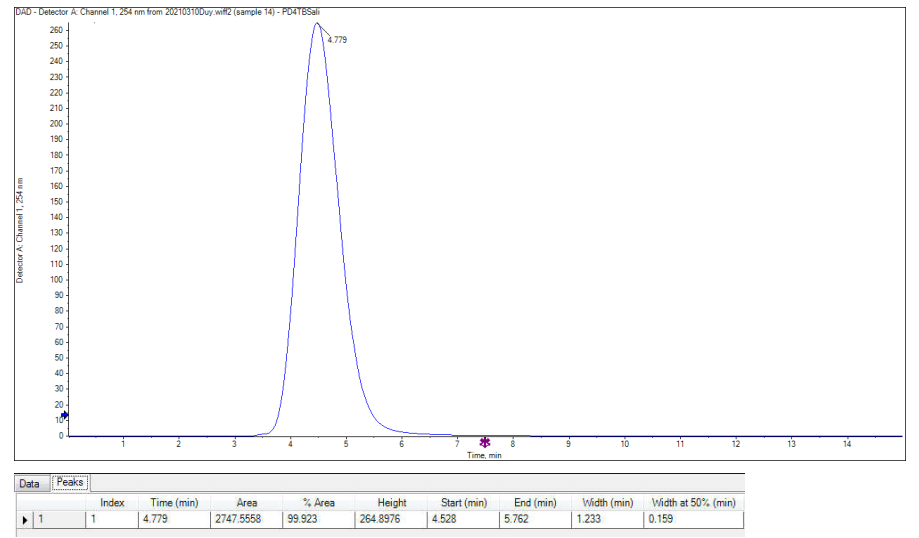


UPLC Spectrum


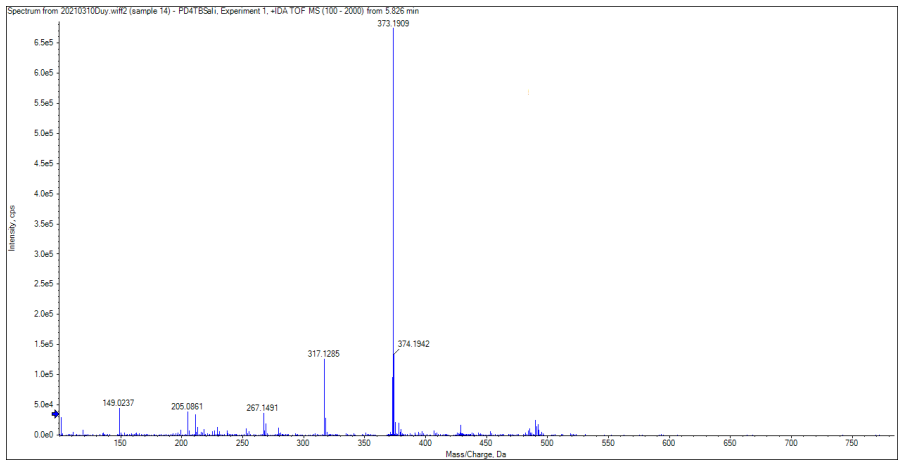


[M+H]^+^

Q-TOF-MS Spectrum


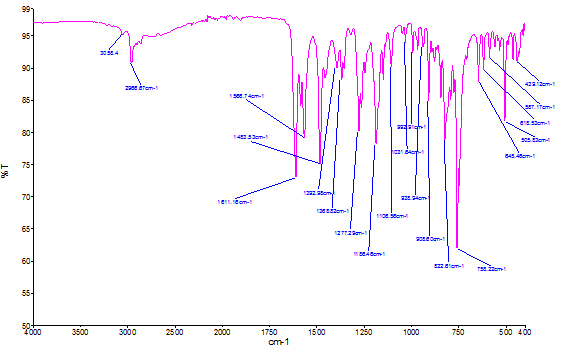


FT-IR Spectrum


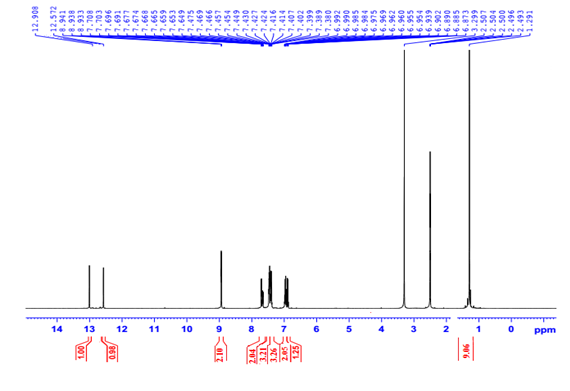


^1^H-NMR Spectrum


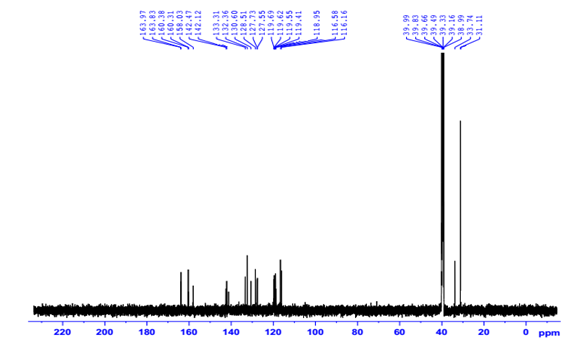


^13^C-NMR Spectrum

I.2. The spectra of ligand H_2_L2


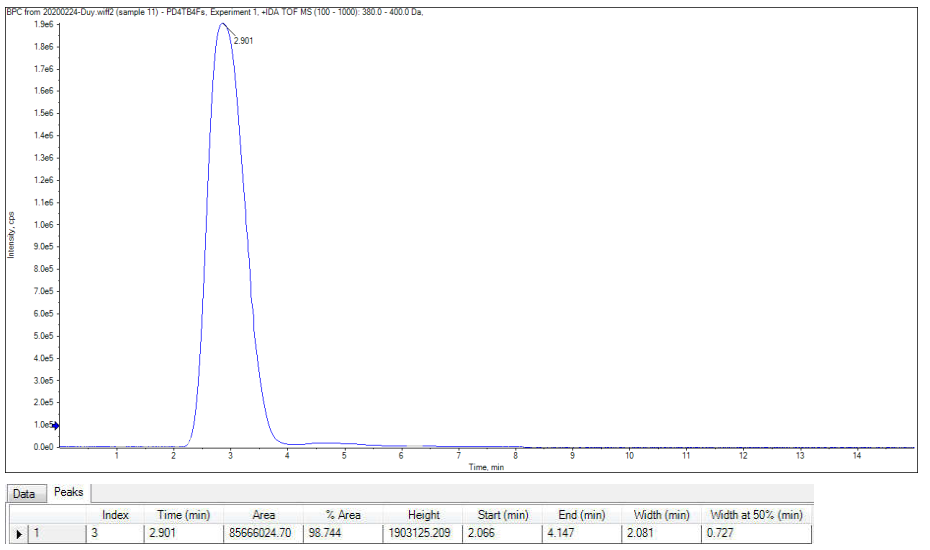


UPLC Spectrum


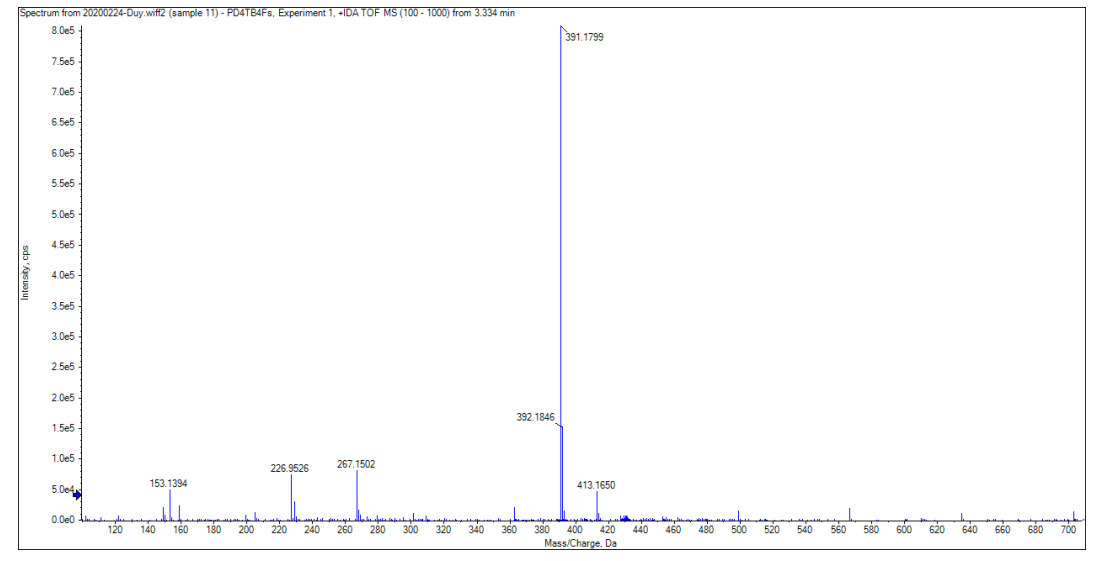


[M+H]^+^

Q-TOF-MS Spectrum


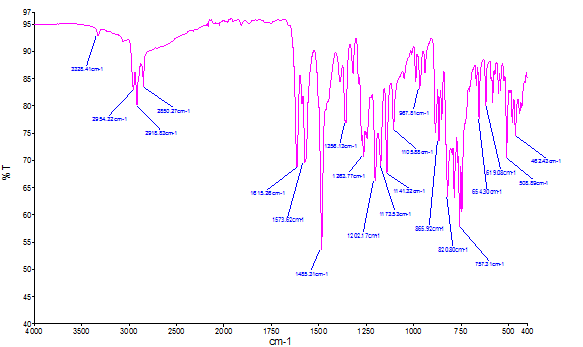


FT-IR Spectrum


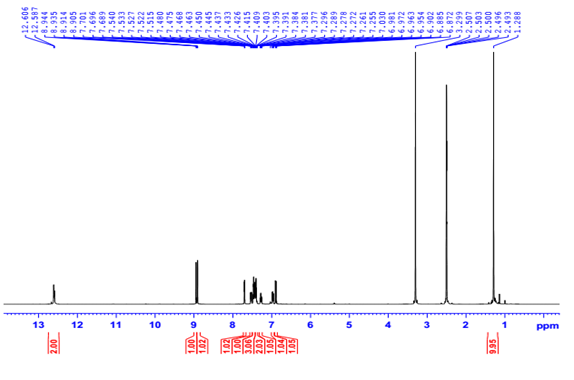


^1^H-NMR Spectrum


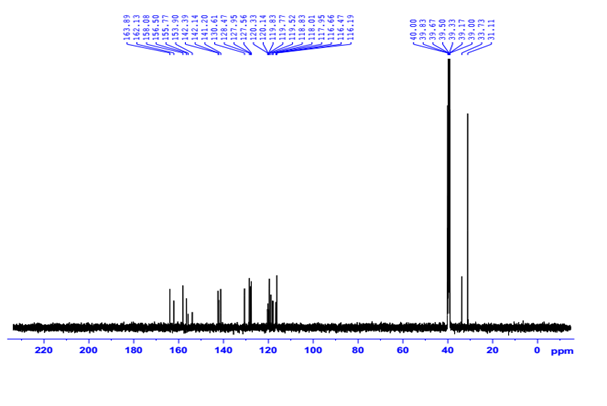
^13^C-NMR Spectrum

I.3. The spectra of ligand H_2_L3


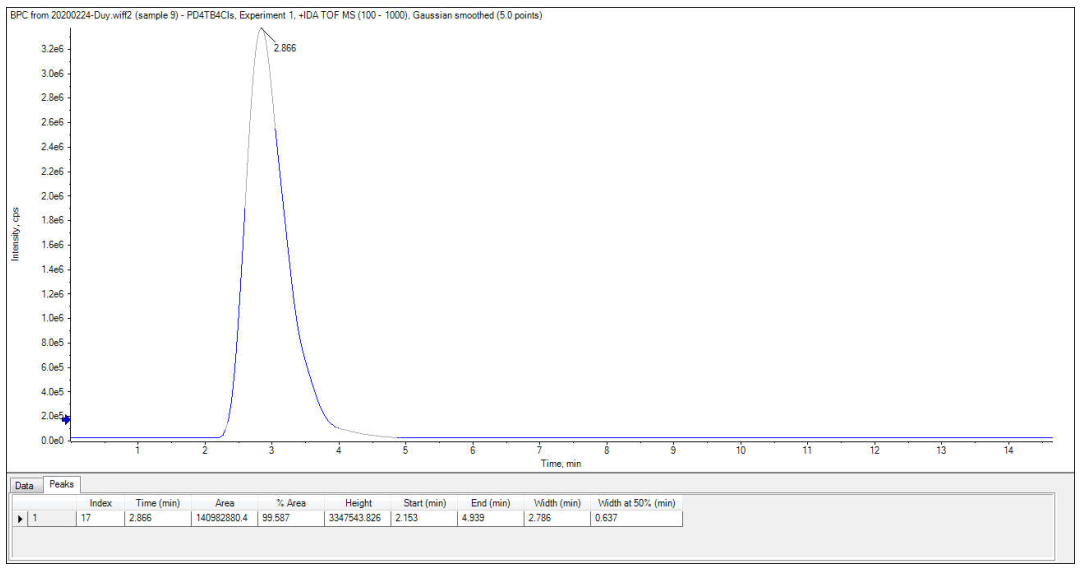


UPLC Spectrum


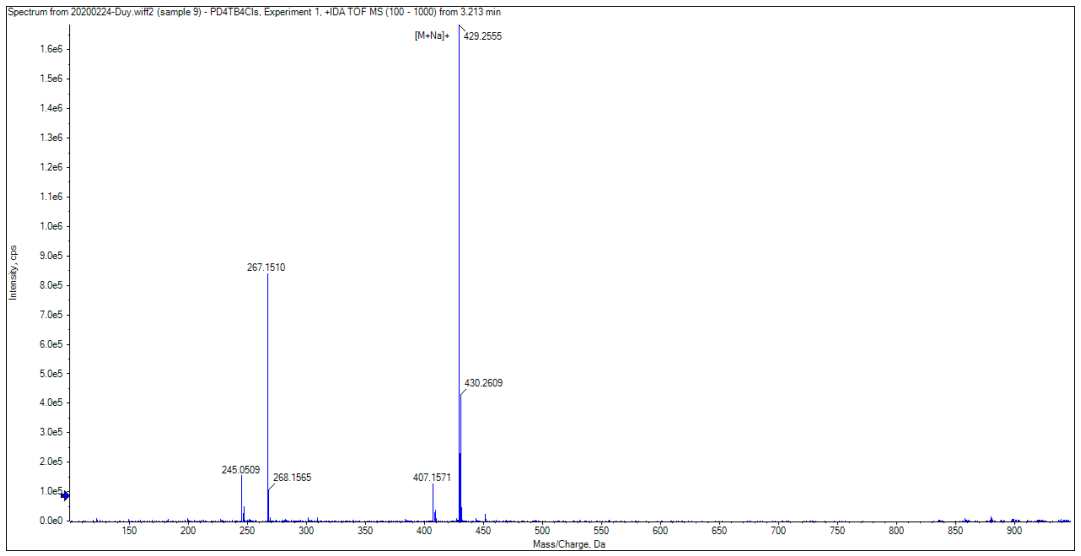


[M+Na]^+^

Q-TOF-MS Spectrum


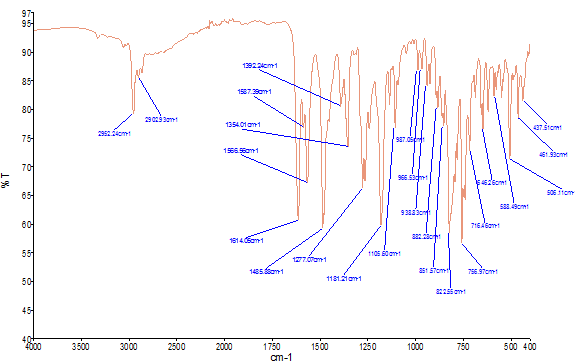


FT-IR Spectrum


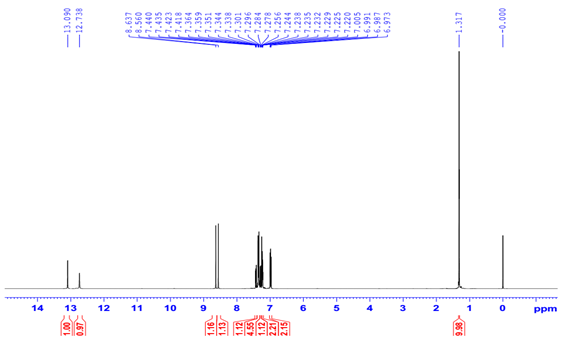


^1^H-NMR Spectrum


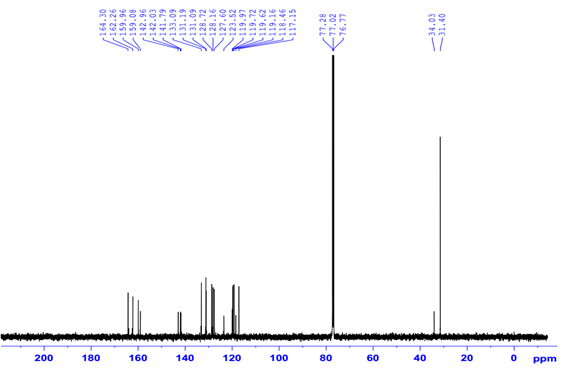


^13^C-NMR Spectrum

I.4. The spectra of ligand H_2_L4


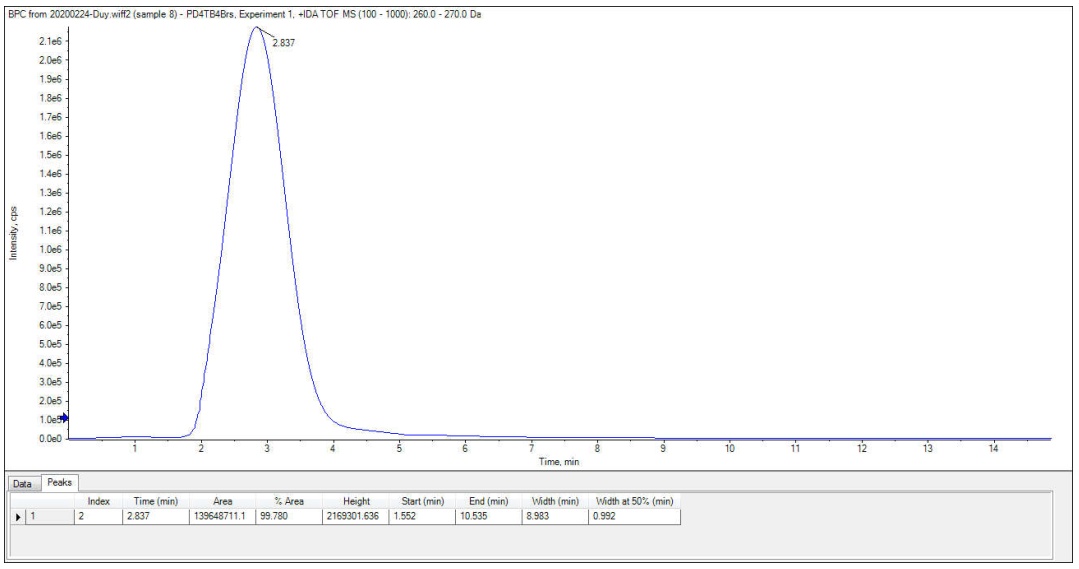


UPLC Spectrum


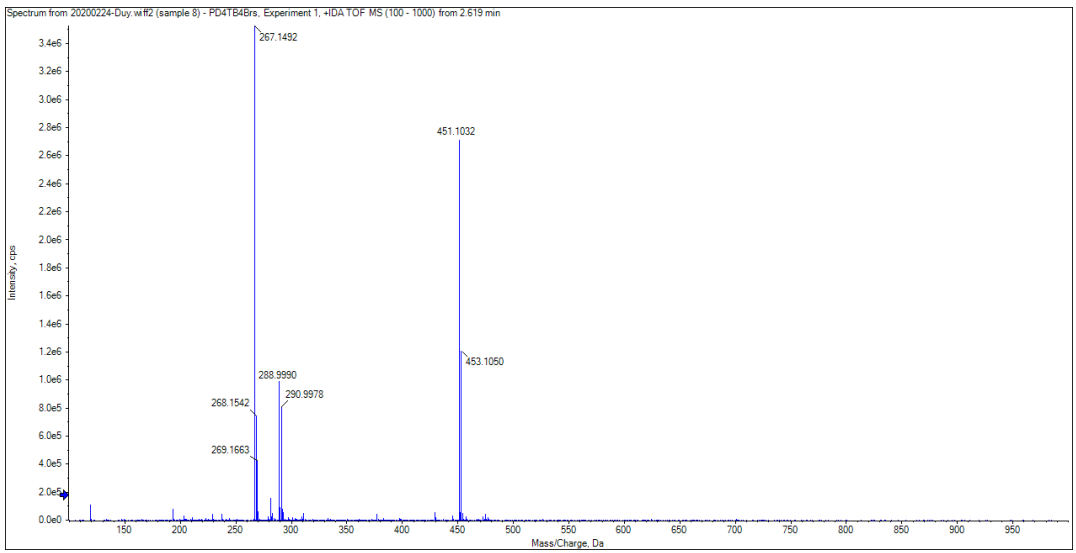


[M+H]^+^

Q-TOF-MS Spectrum


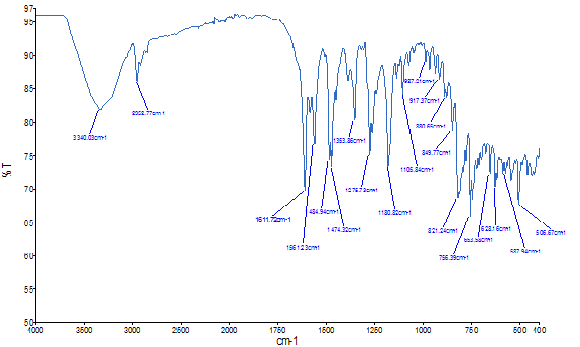


FT-IR Spectrum


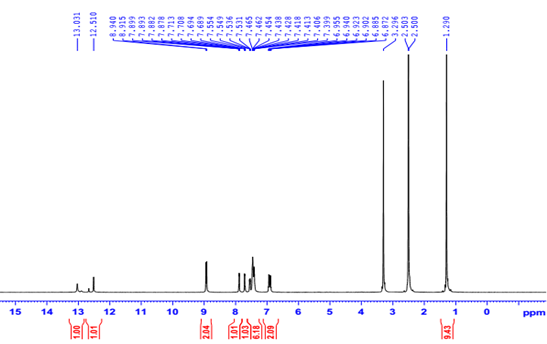


^1^H-NMR Spectrum


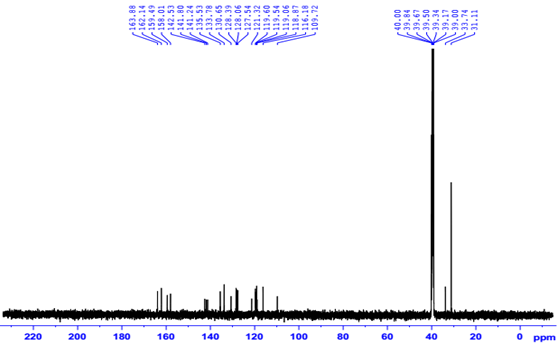


^13^C-NMR Spectrum

I.5. The spectra of ligand H_2_L5


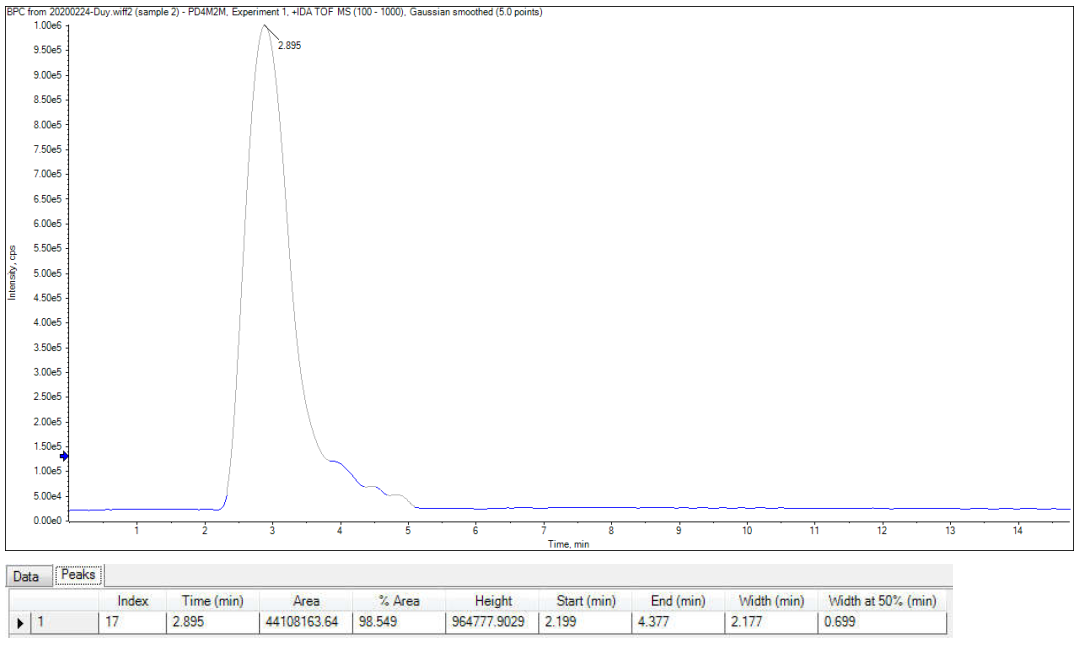


UPLC Spectrum


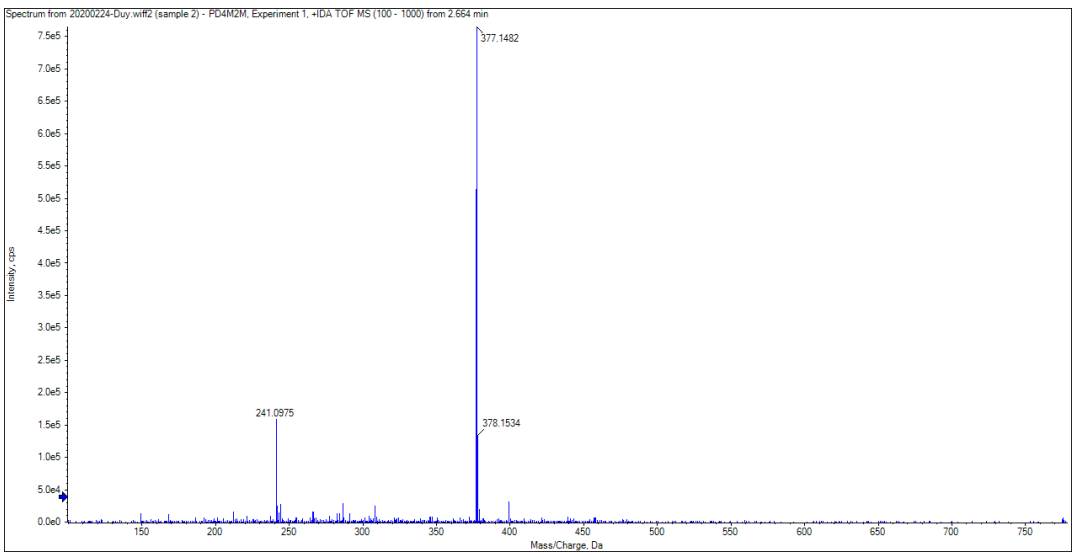


[M+H]^+^

Q-TOF-MS Spectrum


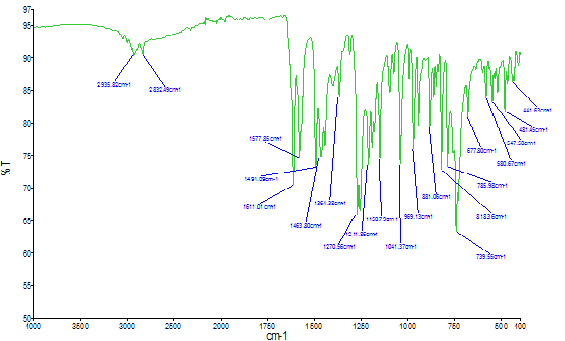


FT-IR Spectrum


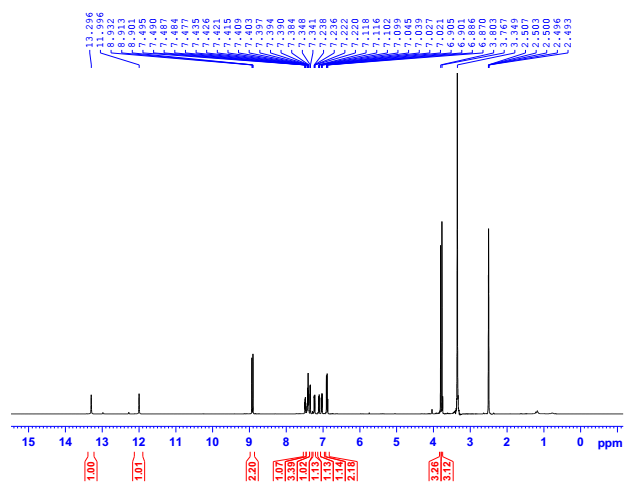


^1^H-NMR Spectrum


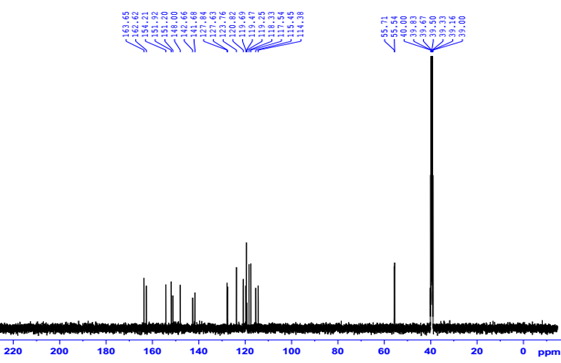


^13^C-NMR Spectrum

1. **The spectroscopies of Cu(II) complexes**

II.1. The spectra of complex [Cu(II)(L1)]


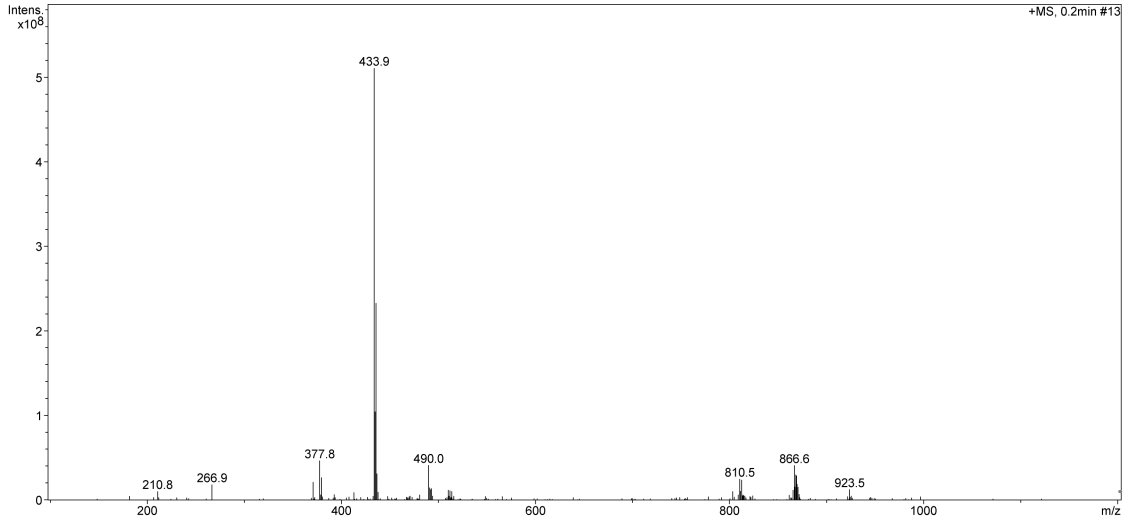


[M+H]^+^

ESI-MS Spectrum


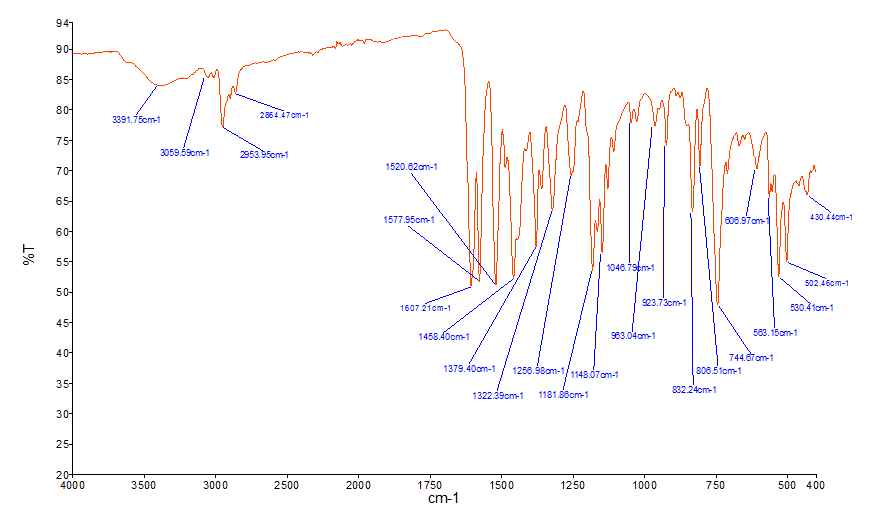


FT-IR Spectrum

CV Spectrum

II.2. The spectra of complex [Cu(II)(L2)]


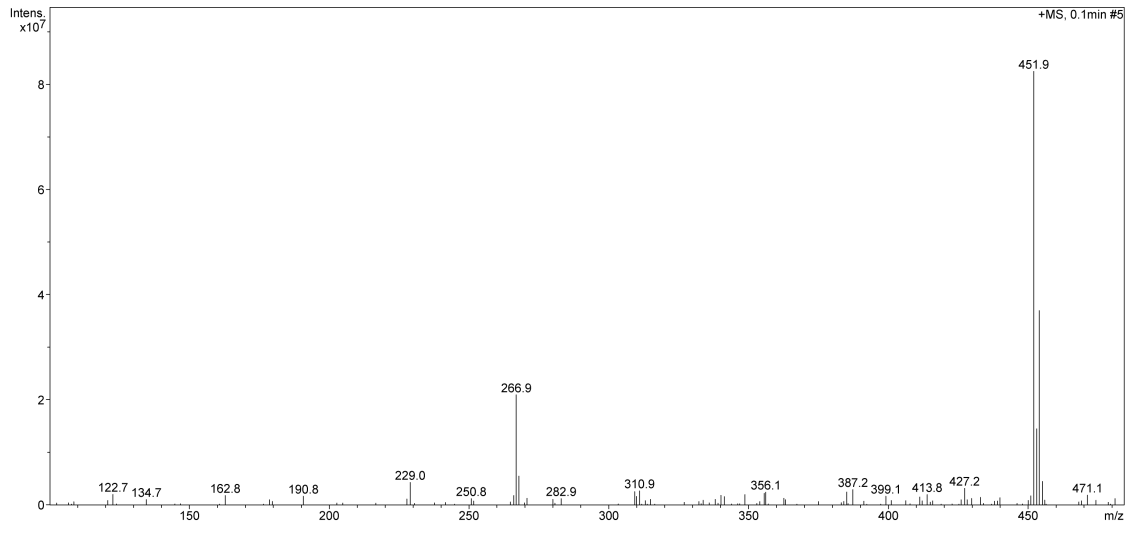


[M+H]^+^

ESI-MS Spectrum


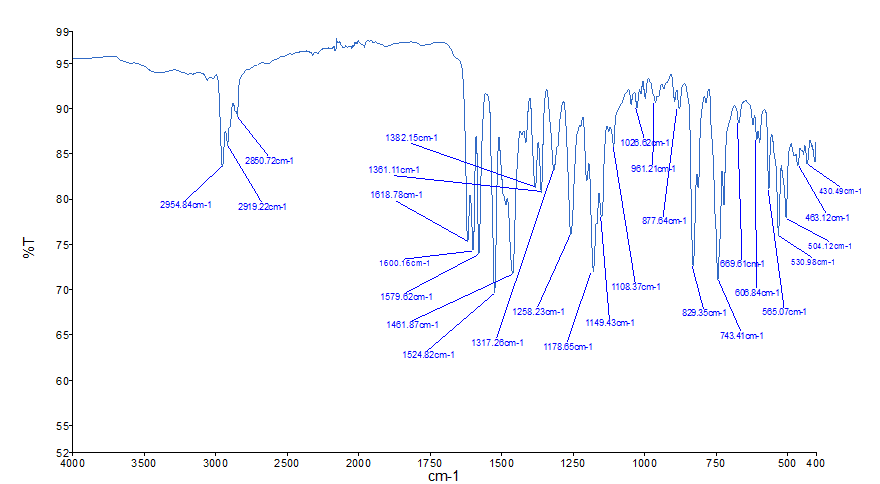


FT-IR Spectrum

CV Spectrum

II.3. The spectra of complex [Cu(II)(L3)]


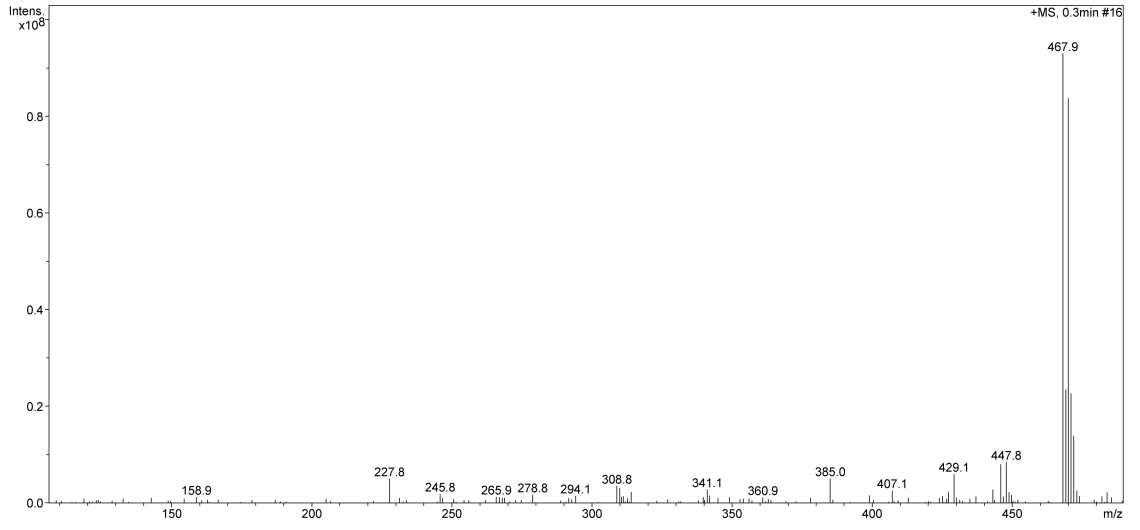


[M+H]^+^

ESI-MS Spectrum


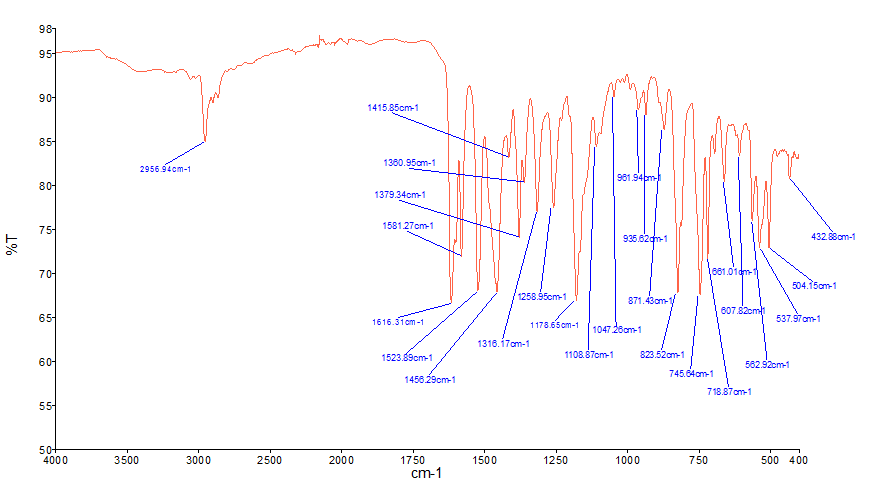


FT-IR Spectrum

CV Spectrum

II.4. The spectra of complex [Cu(II)(L4)]


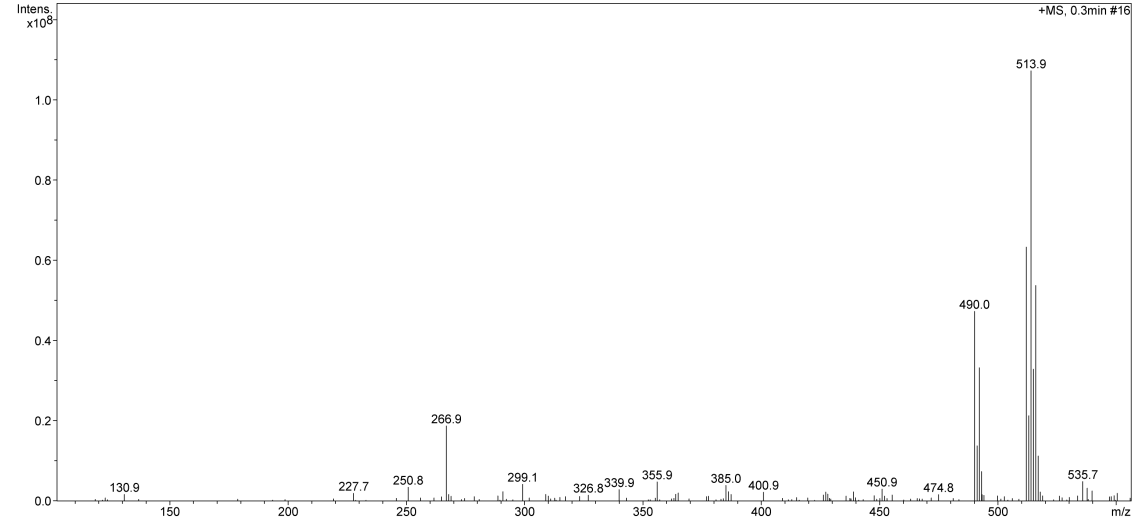


[M+H]^+^

ESI-MS Spectrum


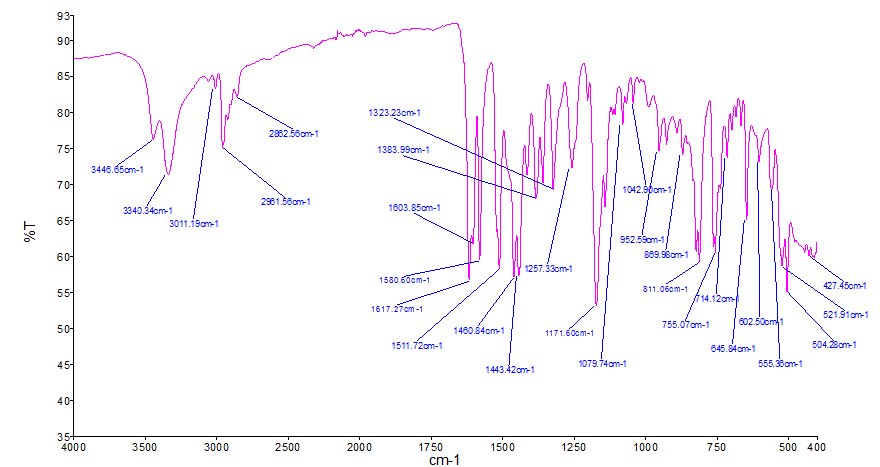


FT-IR Spectrum

CV Spectrum

II.5. The spectra of complex [Cu(II)(L5)]


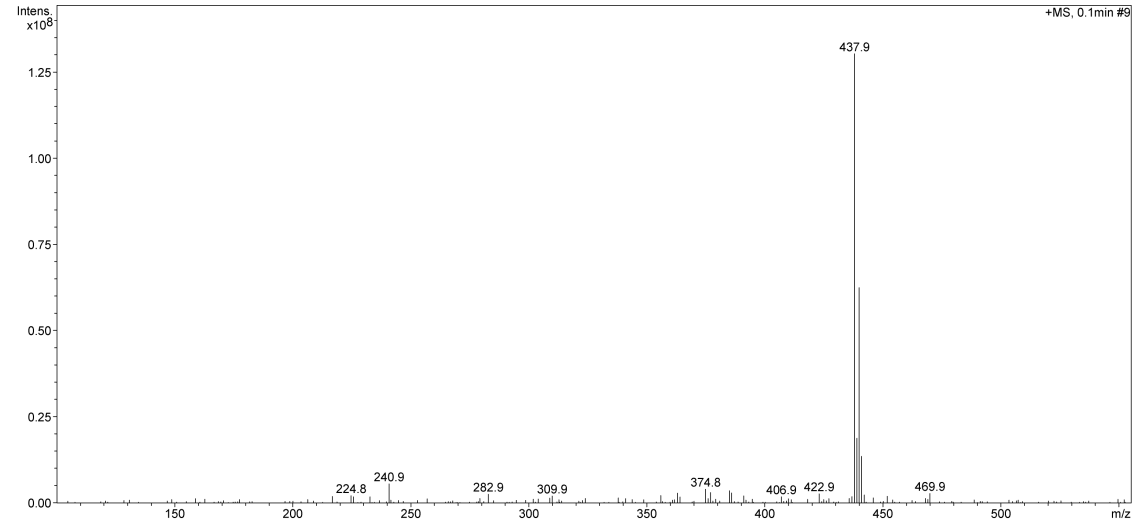


[M+H]^+^

ESI-MS Spectrum


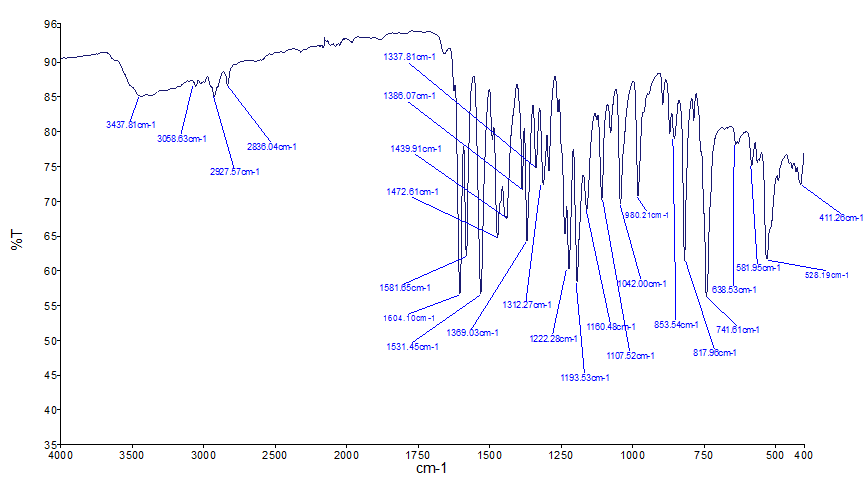


FT-IR Spectrum

CV Spectrum

1. **The spectroscopies of Fe(III) complexes**

III.1. The spectra of complex [Fe(III)L1Cl]


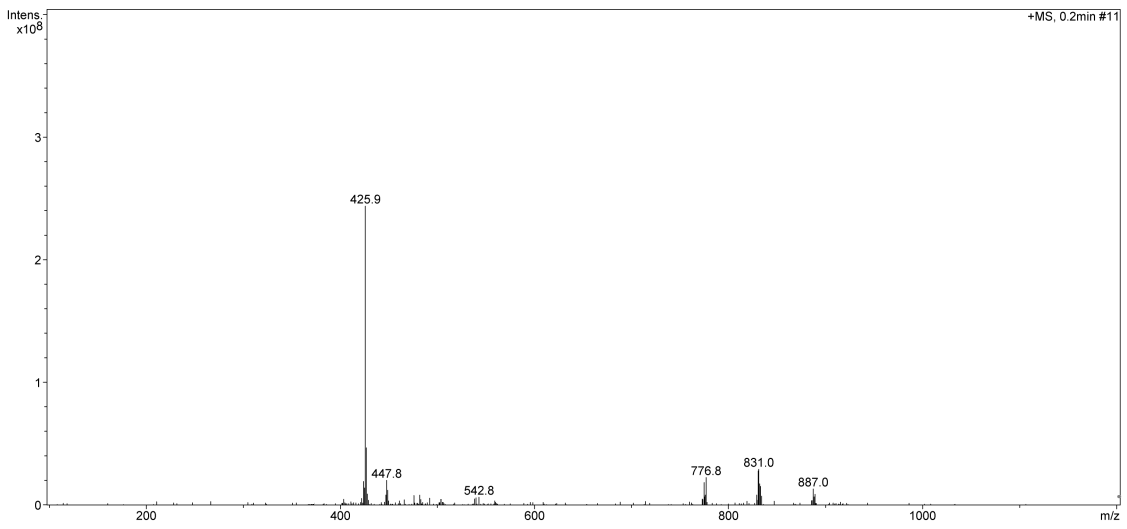


[M-Cl]^−^

ESI-MS Spectrum


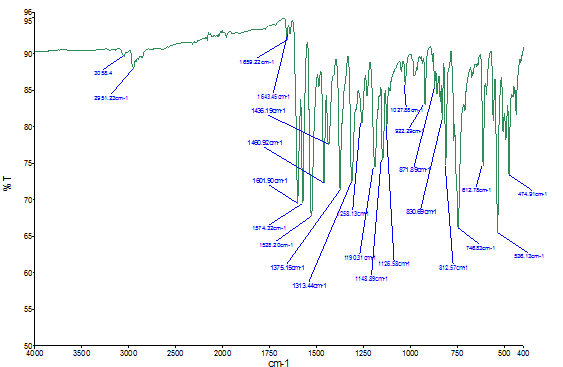


FT-IR Spectrum

CV Spectrum

III.2. The spectra of complex [Fe(III)L2Cl]


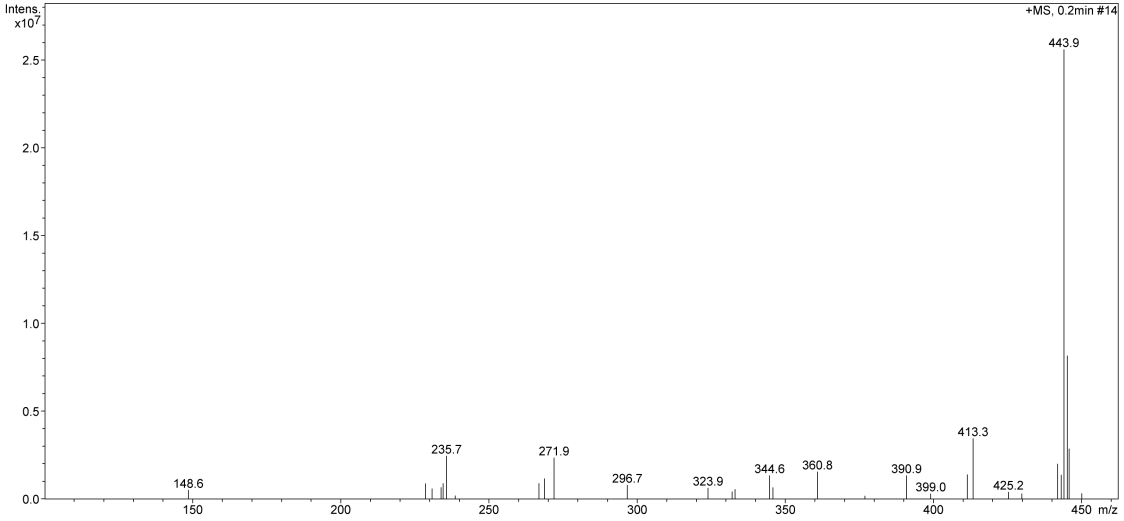


[M−Cl]^−^

ESI-MS Spectrum


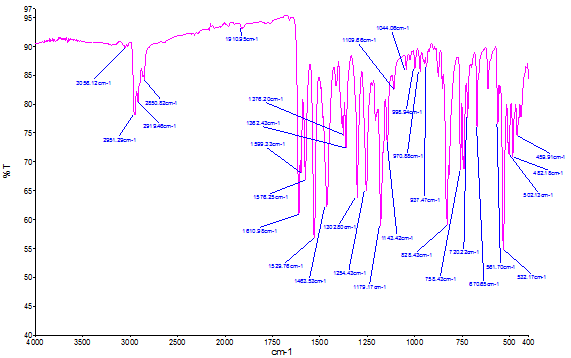


FT-IR Spectrum

CV Spectrum

III.3. The spectra of complex [Fe(III)L3Cl]


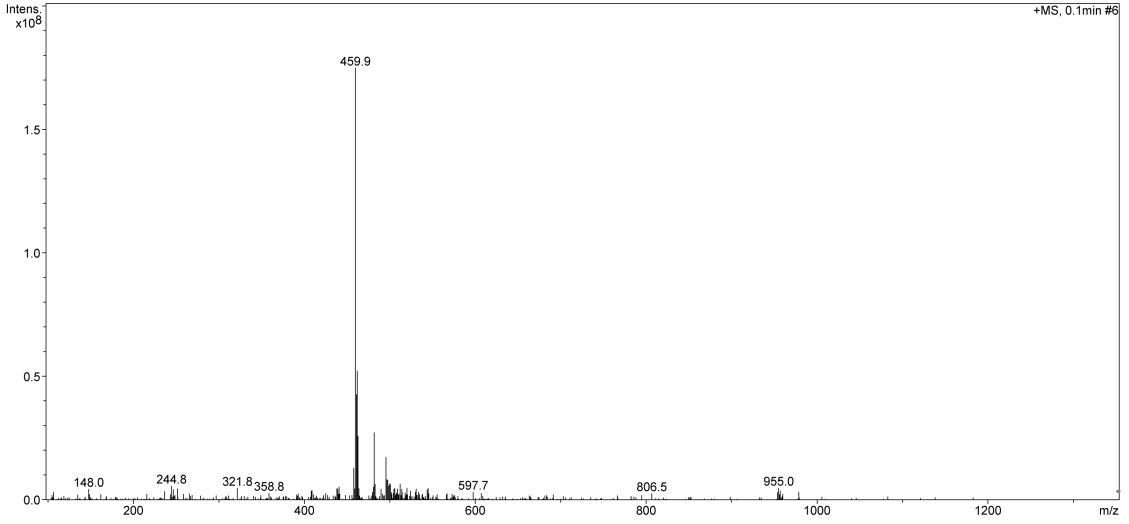


[M−Cl]^−^

ESI-MS Spectrum


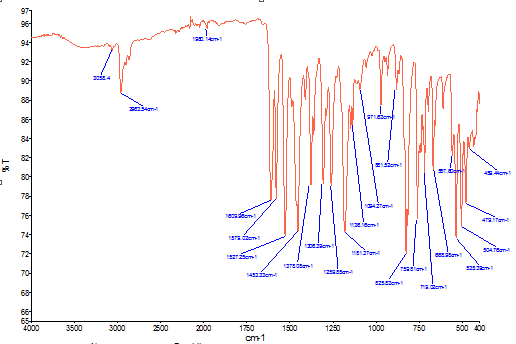


FT-IR Spectrum

CV Spectrum

III.4. The spectra of complex [Fe(III)L4Cl]


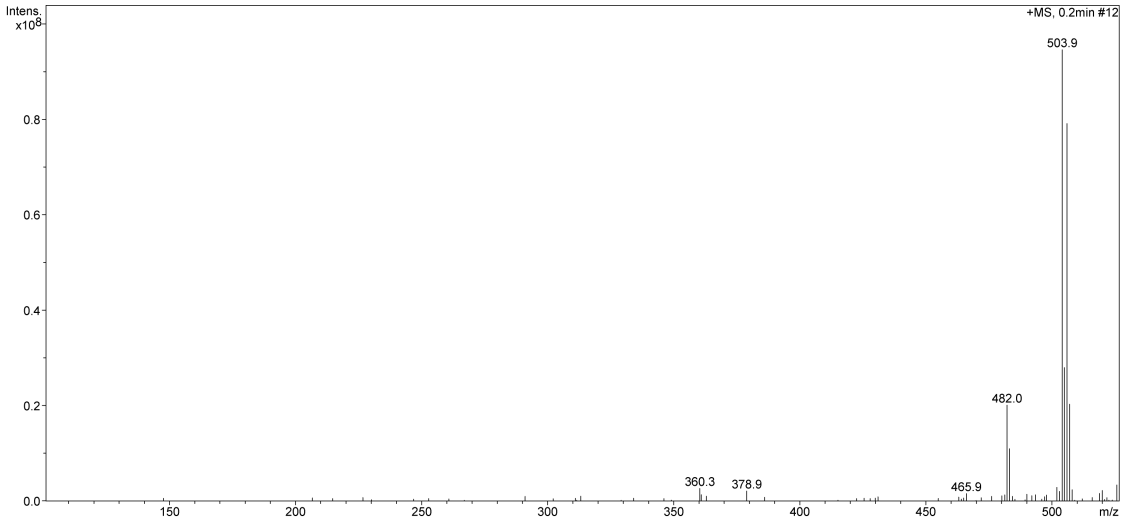


[M−Cl]^−^

ESI-MS Spectrum


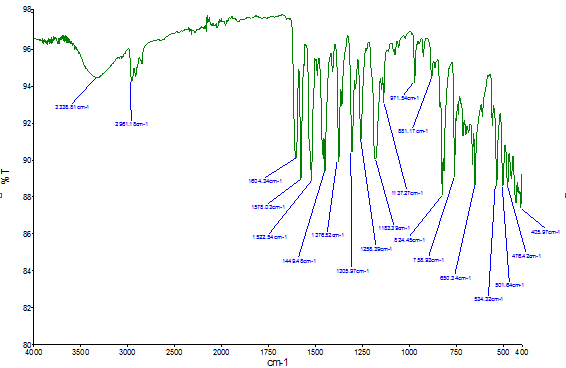


FT-IR Spectrum

CV Spectrum

III.5. The spectra of complex [Fe(III)L5Cl]


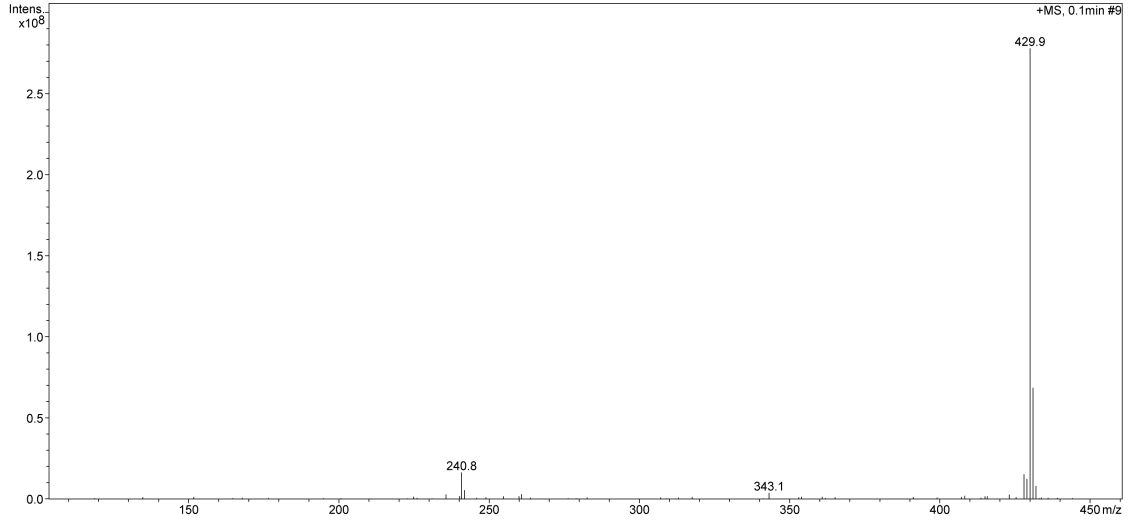


[M−Cl]^−^

ESI-MS Spectrum


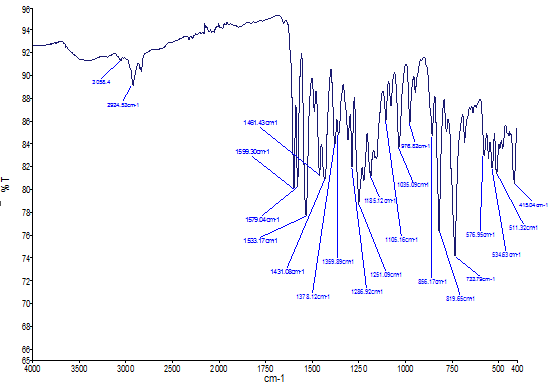


FT-IR Spectrum

CV Spectrum

1. **The UV-Vis spectra of synthetic compounds**

The UV-Vis spectra of H_2_L1 ligand, [Cu(II)L1] and [Fe(III)L1Cl]

The UV-Vis spectra of H_2_L2 ligand, [Cu(II)L2] and [Fe(III)L2Cl]

The UV-Vis spectra of H_2_L3 ligand, [Cu(II)L3] and [Fe(III)L3Cl]

The UV-Vis spectra of H_2_L4 ligand, [Cu(II)L4] and [Fe(III)L4Cl]

The UV-Vis spectra of H_2_L5 ligand, [Cu(II)L5] and [Fe(III)L5Cl]
